# Biomechanical, biophysical and biochemical modulators of cytoskeletal remodelling and emergent stem cell lineage commitment

**DOI:** 10.1038/s42003-022-04320-w

**Published:** 2023-01-19

**Authors:** Vina D. L. Putra, Kristopher A. Kilian, Melissa L. Knothe Tate

**Affiliations:** 1 School of Chemistry and School of Materials Science & Engineering, University of New South Wales, Sydney, NSW Australia; 2 Blue Mountains World Interdisciplinary Innovation Institute (bmwi³), Blue Mountains, NSW Australia

**Keywords:** Cytoskeleton, Stem-cell biotechnology, Differentiation

## Abstract

Across complex, multi-time and -length scale biological systems, redundancy confers robustness and resilience, enabling adaptation and increasing survival under dynamic environmental conditions; this review addresses ubiquitous effects of cytoskeletal remodelling, triggered by biomechanical, biophysical and biochemical cues, on stem cell mechanoadaptation and emergent lineage commitment. The cytoskeleton provides an adaptive structural scaffold to the cell, regulating the emergence of stem cell structure-function relationships during tissue neogenesis, both in prenatal development as well as postnatal healing. Identification and mapping of the mechanical cues conducive to cytoskeletal remodelling and cell adaptation may help to establish environmental contexts that can be used prospectively as translational design specifications to target tissue neogenesis for regenerative medicine. In this review, we summarize findings on cytoskeletal remodelling in the context of tissue neogenesis during early development and postnatal healing, and its relevance in guiding lineage commitment for targeted tissue regeneration. We highlight how cytoskeleton-targeting chemical agents modulate stem cell differentiation and govern responses to mechanical cues in stem cells’ emerging form and function. We further review methods for spatiotemporal visualization and measurement of cytoskeletal remodelling, as well as its effects on the mechanical properties of cells, as a function of adaptation. Research in these areas may facilitate translation of stem cells’ own healing potential and improve the design of materials, therapies, and devices for regenerative medicine.

## Abstract (web summary)

This review highlights the biomechanical, biophysical, and biochemical modulators of cytoskeletal remodeling during tissue neogenesis in early development and postnatal healing for targeted tissue regeneration and regenerative medicine applications.

## Introduction

**Fig. 1:**
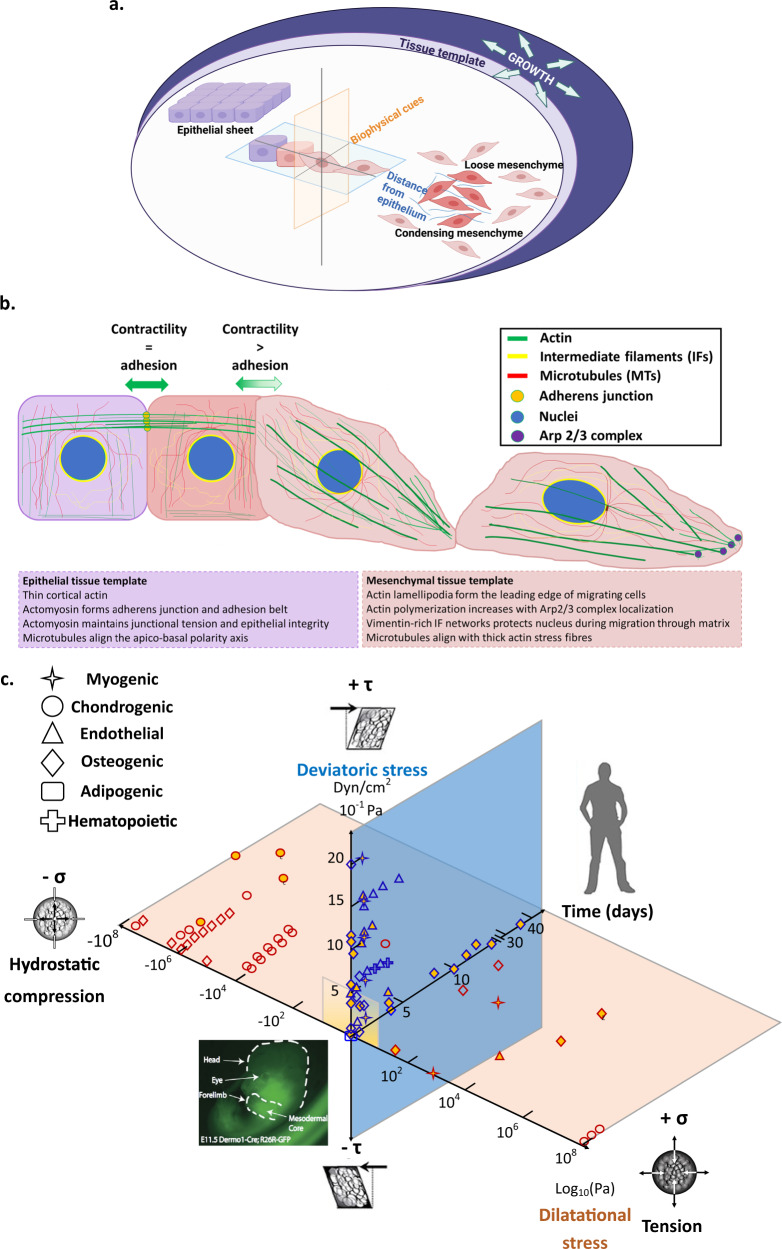
The mechanome, a map of volume (dilational) and shape (deviatoric) changing stresses, relates to exposure to biochemical induction factors, and exposure time, and to lineage commitment (as depicted by data point shape) of stem cells. During prenatal development and postnatal healing, stem cell differentiation and tissue neogenesis are inextricably tied to the mechanadaptation of stem cells, which can be observed in the remodeling of the actin and microtubule cytoskeleton^4^. **a** The actin and microtubule cytoskeleton remodel in response to the spatiotemporal presentation of biophysical and biochemical cues within the tissue. The transition between epithelial (sheet-like) and mesenchymal (globular) tissue templates is itself mechanically modulated and enables the growth and specialization of tissue structure and function (adapted with permission from ref. ^1^). **b** Cytoskeletal remodeling underpins the epithelial-to-mesenchymal transition (EMT), including the formation of lamellipodia and the rapid polymerization of parallel stress fibers, resulting in front–back polarity of the mesenchymal cell, itself a mechanoadaption. The remodeling is necessary to drive EMT from tensional balance between epithelial cells where adhesion force equals contractile force, to higher contractility at the interface of ingressing and non-ingressing cells. **c** The mechanome map of the cells’ actual stress–strain data upon introduction of mechanical and biochemical cues and the corresponding fates. Cells experience intrinsic mechanical cues during development such as volume-changing (dilatational) stress (red plane, *x* axis) (i.e., hydrostatic compression and tension [log_10_ Pa]) and shape-changing shear stress (blue plane, *y* axis) (shear stress magnitudes [dyn/cm^2^ or 0.1 Pa], that dictate their lineage commitment over time (*z* axis) (adapted with permission from ref. ^82^, ref. ^2^). Stem cells’ emergent mechano- and differentiation responses, presented as different shape data points, demonstrate the spatiotemporal adaptation to the magnitude of mechanical cues and the interplay with biochemical cues (i.e., induction medium, indicated by orange filled data point shapes). The yellow plane with opaque to transparent gradient represent the ranges of stress predicted during early development in utero^5,6,17,20,21,117,125,169–201^.

The lifelong processes of prenatal development, growth, and postnatal healing are mechanobiological^1–3^. Throughout these processes, cells continuously adapt to their changing mechanical *milieux*; cellular mechanoadaptation underpins the emergence of structure–function relationships within cells and the tissue templates (*Anlagen*) they build, the organs that tissues form, and the organisms comprising systems of organs (Fig. 1a)^1,4^. The biophysical cues intrinsic to the cell’s environment shape the resultant, emergent biological structures^5^, presenting a mechanical feedback loop whereby stem cells regulate the synthesis and degradation of extracellular matrix (ECM, outside the cell), and the polymerization and depolymerization of their cytoskeletal filaments (cytoskeleton, within the cell)^4,6,7^. The capacity of cells to undergo unique and adaptive cytoskeletal remodeling for prevailing environmental conditions is seminal to the emergence of living architectures at higher length scales (Fig. 1b). This capacity is ubiquitous amongst species and cell types, and the redundant mechanisms underpinning cytoskeletal remodeling and subsequent mechanoadaptation of cells result in increased resilience and survival of cells in dynamic environments^8–10^. Elucidation of this cellular capacity will translate to design criteria in support of tissue engineering and regenerative medicine endeavors, e.g., enabling proactive steering of targeted of stem cell differentiation and creation of tissue templates emulating specific, targeted developmental stages^4^.

The cytoskeleton, a network of filamentous polymers, confers structural integrity to cells and regulates essential biological function in the cells’ mechanically dynamic environment, e.g., motility, “growing into” the environment, and lineage commitment. Consisting of actin microfilaments, intermediate filaments (IFs) and microtubules, each element of the cytoskeletal network performs unique mechanical roles which coordinate to enable integrative cellular function. Of all cytoskeletal filaments, only actin and microtubules are polar, i.e., they polymerize at one end and depolymerize at the other, which imparts directionality (necessary for vector function in a physics context) to their behavior. Combined with this polarity, the changing length of actin provides contractility and tension-sensing capacity to cells^11^. The ubiquity and the highly conserved sequence of the actin protein across eukaryotic cells underpin cell behaviors as varied as “exploration” and “growing into” environments (e.g. formation of filipodia, lamellopodia and membrane ruffles), transport of organelles, maintenance of cell shape, and cell motility^12^, IFs protect the cells’ architecture and nuclear integrity^13^, support organelles’ position within the cytoplasm^14^, and facilitate crosstalk between actin and microtubules^15^. Microtubules generate the push-pull forces for directional transport across the cytoplasm and provide resistance to compression^16^. Beyond the direct mechanical roles of cytoskeletal elements in scaffolding the cell and physically connecting the cell to other cells and matrix, the remodeling of actin^6,17,18^, microtubules^6,7,19^, and IFs^20^ under mechanical stimuli (e.g. induced by seeding protocol and density^6,21^, mechanical loading^17^, and vibration^18^ at different length and time scales), modulates the interaction between cytoskeleton and proteins that regulate dynamic cytoskeletal behaviors^22,23^ and mechanoadaptation itself.

Unlike an engine and its parts which are designed for highly specific and efficient function, the remodeling and motility of cells emerge from the arrangement of their molecular “parts” over time. The so-called “molecular clutch hypothesis”^24,25^, introduced more than two decades ago, describes the emergent cellular behavior resulting from force balances at and below the length scale of the cell itself, i.e. a metaphoric clutch engaging with the local substrate in a stiffness-specific manner. In short, “when the clutch is engaged—that is, the actin flow is coupled to the ECM through focal adhesion (FA) proteins and immobilized integrins—the force due to actin polymerization would result in slowing down of the retrograde flow, protrusion of the leading edge, and generation of rearward traction forces by which the cell can be propelled forward”^25^.

The molecular clutch is hypothesized to enable cells’ capacity to “sense” the relative stiffness of their local mechanical environment and provide a molecular mechanism for emergent tissue length-scale behaviors such as motility, growing into the local environment^24–26^ and thereby epithelial-mesenchymal transitions (Fig. 1b); over time this emergent behavior provides molecular underpinnings for tissue adaptation to prevailing stresses^4^.

The cumulative mechanoadaptation of cytoskeletal elements to complex, dynamic physiological cues culminates in lineage commitment^4^. In this way multipotent cells differentiate towards a steady state with structural specialization appropriate for the cells’ unique, prevailing environment^4^. The central mechanism by which cells “know” to remodel their structure, placing structural materials in the appropriate places for optimal function, remains unknown^4^. Genetic knockdown approaches have shed some light on these processes, but given the integrative nature of the cell, such “top-down” approaches are expected to exert multifaceted effects on cellular behaviors. There is a need for “bottom-up” approaches to decipher mechanisms of cell mechanoadaptation. Cytoskeletal modulating agents can be used as tools to decouple the complex processes of dynamic cytoskeletal protein de-/polymerization. For example, chemical agents including cytochalasin D and paclitaxel respectively induce actin filament aggregation and/or branching, which in turn literally shape the cell and regulates lineage commitment^5,21,27^. For example, actin branching has been shown to be necessary for osteogenesis and preventing such branching favors adipogenesis^28^. The study of stem cell mechanoadaptation in controlled contexts using cytoskeletal modulating agents offers new opportunities to decipher the role of mechanoadaptation in differentiation. In turn, application of this knowledge in context of materials development and design, e.g. biophysically modulating topography^29^, stiffness^30^, porosity^31^ and/or architectures, will provide reference points for integrated design of mechano- and bioactive implants and replacement body parts (i.e., bionics)^32,33^.

Here we review

- the regulation of cytoskeletal remodeling, focusing on actin and microtubules, as a function of stem cell mechanoadaptation during tissue development and healing,
- mechanical and chemical modulation of cytoskeleton for targeted differentiation and tissue neogenesis; and
- quantitative imaging methods for spatiotemporal visualization and measurement of cytoskeleton adaptation.

We explore how engineered materials target cytoskeletal remodeling and guide multiscale developmental responses, particularly through changes in actin and microtubule dynamic de-/polymerization, spatial distribution, and mechanics. Elucidation of cytoskeletal adaptation across time and length scales will provide a bottom-up basis from which to understand collective cellular behavior, e.g., in tissues, organs, and organ systems. Elucidation of the redundant roles of mechanical, chemical, and biophysical cues on cell mechanics and behavior, referred to as “mechanomics” (Fig. 1c and Box 1), underpinning stem cells’ own regenerative power, will provide critical design considerations for next-generation materials, therapeutics, implants, and medical devices.

Box 1 Literature reports of stem cell mechanoadaptation, linking cytoskeleton remodeling to mechanically and biochemically induced differentiation

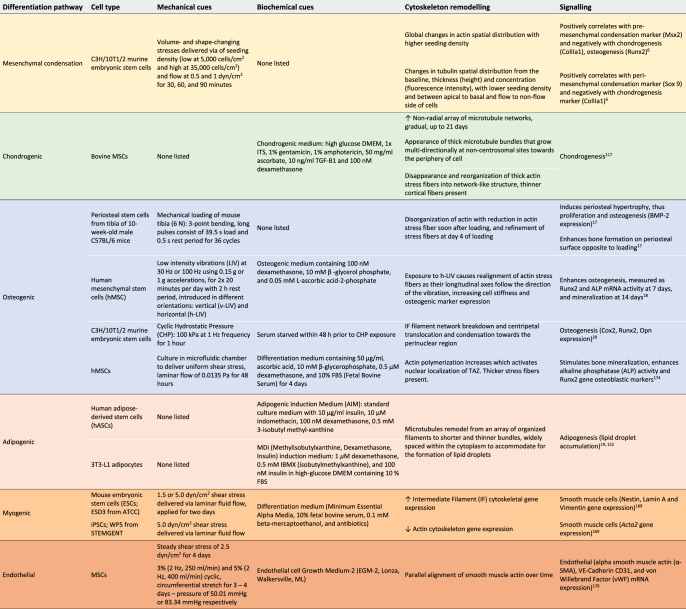

## Cytoskeleton remodeling as a mechanoadaptative process

### Actin remodeling and focal adhesion dis-/assembly

**Fig. 2:**
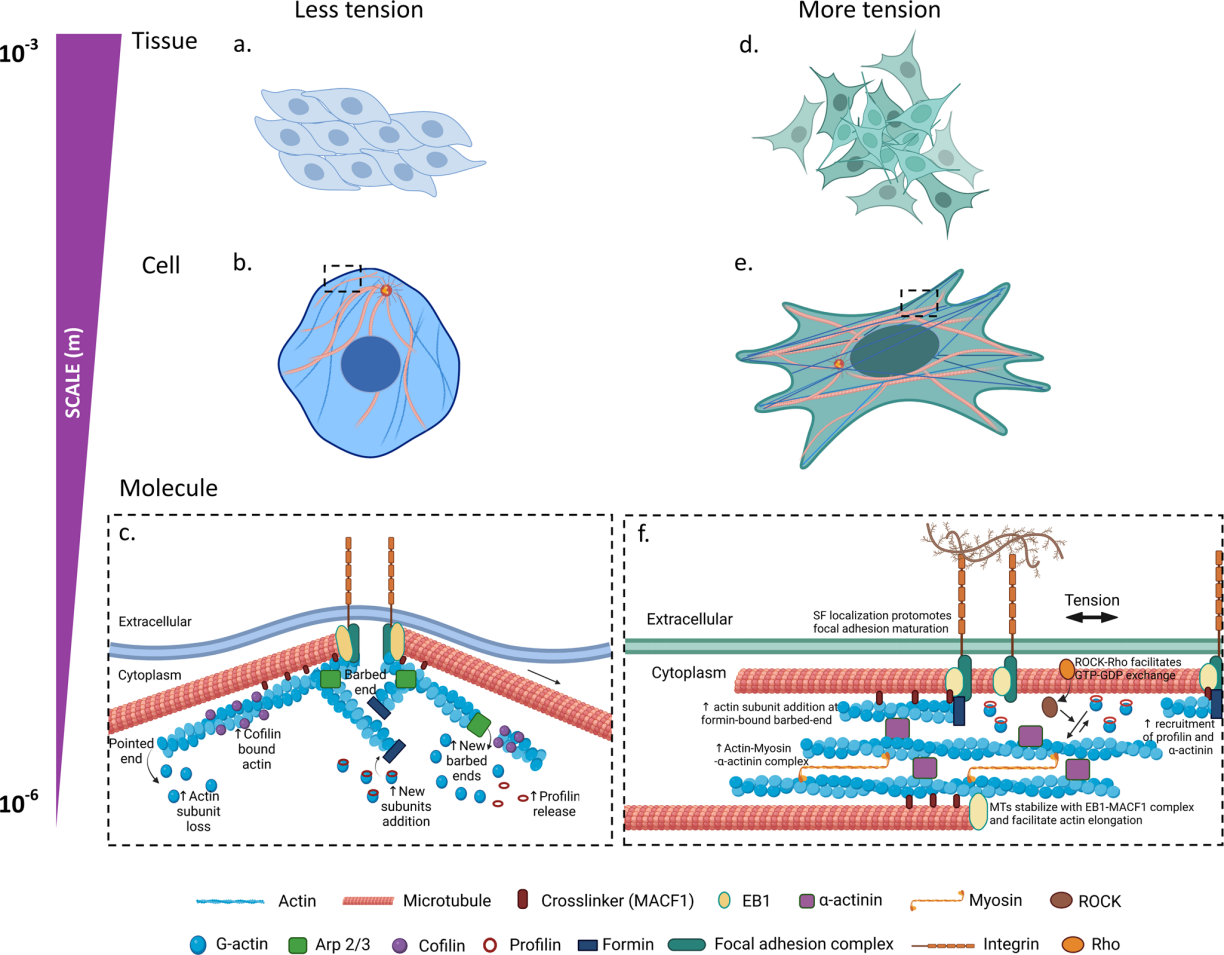
The remodeling of actin and microtubules due to tensional cues delivered as stretch or barriers to stretch via substrate rigidity, and their multiscale translation to the structural and mechanical properties of cells and tissues. Culture of tissue on soft substrates (**a**) reduces the intrinsic tension of stress fibers (SF) and thus increases cell relaxation (**b**); this in turn permits cofilin binding to actin and promotes actin disassembly or severing of actin (**c**). Cofilin binding increases the rate of actin subunit loss from the pointed end of actin filament, releases profilin from the filament and increases the number of free barbed ends where subunits can be added. The growing ends of microtubules sense the periphery of cell and thus maintain filament growth. Microtubules do not necessarily target focal adhesion, but their end-binding protein (EB1) forms complexes with microtubule actin cross-linking factor 1, MACF1, that is enriched at focal adhesion complex. Increased tension due to culture on stiff substrates (**d**) increases intrinsic tensile stress of the cell, and thus actin reinforcement (**e**). Tension enhances actin polymerization as it recruits profilin and α-actinin actin polymerization factors (**f**). The formin FH2 domain-bound barbed end of actin enhances the rapid addition of actin subunit-bound profilin via interaction with the formin FH1 domain. SF localization provides templates for focal adhesion maturation, reduces the pool of free G-actin monomers, and inhibits binding of cofilin. ROCK and Rho signaling facilitates GTP to GDP exchange for actin polymerization. Actin forms thicker SF bundles via complexes with myosin and α-actinin to adapt to increasing tension. Tension stabilizes microtubule structure and promotes alignment with actin in the direction of tension. Microtubule growth facilitates SF assembly and elongation via transport of actin polymerase proteins.

But for the cytoskeleton, cells could not adapt to their dynamic mechanical environment. Mechanoadaptation is essential to cell survival in dynamic mechanical environments. Actin serves as the main mechanoresponsive filament of the cytoskeleton, linking the cell’s organelles and cytoplasm to the extracellular matrix (ECM) via integrin-mediated focal adhesions (FAs)^34^, and to other cells via cell–cell adhesions (e.g., E-cadherins, selectins) (Fig. 2).

Cell–cell and cell–matrix adhesion dynamics: The formation of cell–matrix and cell–cell adhesions is itself mechanically modulated and represents a composite structure, like a bird’s nest, formed by the interaction of myriad mechanically modulated “parts” (e.g., actin cap associated focal adhesions, ACAFA)^35^. Actin stress fibers (SFs), with periodically arranged myosin II (actomyosin), provide long-distance connectivity and propagate forces between focal adhesions and the nucleus, as well as across the cytoplasm, through their contractility^36^. The focal adhesion (FA) complex consists of Talin (the major linker protein between actin and the ECM), Vinculin, Paxillin, Vasodilator-stimulated phosphoprotein (VASP), focal adhesion kinase (FAK), and p130Cas, all of which perform mechanotransduction functions and regulate actin dynamics (de-/polymerization), bundling, and thus force generation^37^ (Fig. 2).

While “brainless” cells cannot sense and measure the absolute stiffness of substrates to which they adhere, mismatches or relative differences in stiffnesses at their boundaries regulate force balances between cells and their local environments. If forces are not balanced, then displacements or movement results. Hence, stiffness mismatches between cells and their substrates, drive not only mechanoadaptation but also motility^4,38,39^. Cells exhibit a range of stiffnesses on the order of 0.1–20 kPa, depending on their source (e.g., cancer cell lines, which are softer), cytoskeletal characteristics^40,41^, and motility state. During adaptation to such relative stiffness differences, actomyosin contractility generates traction force in adherent cells.

Focal adhesion dis-/assembly: As noted above, actomyosin is arranged periodically within SFs. FA assembly depends on the magnitude of the traction force and depends on stiffness mismatch between cells and their surroundings^42^. For example, with increasing substrate stiffness (e.g., 3–14 kPa), mouse embryonic fibroblasts (MEFs) increase traction force and improve localization of vinculin, an actin-binding protein, to FA^43^; inhibition of contractility via treatment with the myosin II inhibitor blebbistatin significantly reduces vinculin localization to FA and decreases FA area on stiffer substrates (5–14 kPa)^43^. However, for MEFs expressing a vinculin mutant with open conformation that readily binds talin and actin (vinculin-T12)^44^, both vinculin-T12 and paxillin exhibit significant localization to FA across soft and stiff substrates, with vinculin residence time depending linearly on stiff substrate and while paxillin residence time depending on softer substrates^43^. This further substantiates the molecular clutch hypothesis^25^ i.e. that actin and FA complex dynamics operate as a function of stiffness mismatches; notably cells and their mechanoresponsive elements readily respond to a range of substrate stiffnesses above a certain threshold.

During morphogenesis, wound healing, and tumor progression, the molecular clutch dynamic is central for cell mechanoadaptation to ever-changing mechanical properties of tissues (e.g., rigidity or stiffness)^25,45–48^. As mentioned previously, engagement of the molecular clutch is stiffness-dependent. On stiff substrates, actin-clutch engagement is short-lived; due to rapid tension build-up against stiff ECM, actin retrograde flow (the centripetal movement of actin filament) rate increases and mean traction force decreases^38^. On softer substrates, the slower tension build-up allows the clutch engagement to persist below its breaking strength, in turn slowing actin retrograde flow and enabling larger transmission of traction force^38^.

Investigation of filopodial traction forces in embryonic chick forebrain neuron cells cultured on soft (730 Pa) polyacrylamide gel reveal that individual filopodia show variable, region-dependent responses and adapt by load-and-fail dynamics—where loading occurs as the filopodia move toward the growth cone and failure commences as they revert to resting state, followed immediately with load within 100-ms time intervals^38^. With stiffer gels (from 57 kPa), such failure event occurs more frequently^38^; this corroborates models predicting actin-talin bond failure and the increasing likelihood of clutch rupture with increasing substrate stiffness^40^. The balance and trade-off between clutch rupture and stability underpins cells’ capacity to adapt to a wide range of stiffness in their immediate environment^40^.

A further subcellular mechanoadaptation mechanism works through an increase in the area of focal contact adhesions, to a steady-state size in proportion with elastic compliance over a time scale^49,50^. Consistently, experiments testing the molecular clutch hypothesis have confirmed that the rate of force and/or loading exposure initiate mechanosensing and cytoskeletal softening^51^, i.e., cytoskeletal remodeling. Higher loading magnitudes up to 20% stretch and loading rates below 1 Hz increase YAP (yes-associated protein) nuclear translocation and paxillin growth in MEFs. However, at higher loading rates, increased loading magnitudes decrease mechanosensing concomitant to actin softening, in effect stabilizing the response^51^.

### Role of Talin stretch in the formation of focal adhesion complexes

As noted above, the assembly of FAs is modulated both by cell traction force magnitude as well as the mismatch in stiffness between cells and their surroundings. This mechanical modulation extends down to the molecular length scale. As the major linker protein between actin and the ECM, Talin’s capacity to stretch influences both its bind affinity for vinculin (and form FACs) at a molecular length scale as well as its capacity to act as a mechanical buffer (elastic element or damper) between the cell and its matrix at the cellular length scale. By mediating effective force transduction to and from the substrate via the unfolding or stretching of Talin, the molecular clutch becomes highly engaged with increasing substrate rigidity above a threshold where Talin is maximally stretched and is also in a state most conducive to binding vinculin. Below this threshold, the force is released through unbinding of integrin^26^. Specific stiffness and threshold values for this balance in FA formation and unbinding of integrin depend on specific cellular contexts, e.g. defined by cell type, cell environment in situ (in vivo or in vitro), including endogenous and exogenous mechanical state, and the mechanical state of linker proteins between the cell and the ECM. Integrating knowledge gained from a diverse experiments and experimental conditions, as well as using diverse computational, mathematical, and experimental models, will enable future extension of the mechanome map (Fig. 1) to the subcellular length scales relevant for the molecular clutch.

Despite the known force-dependency of Talin and Vinculin interactions^52,53^, it is possible for Talin to bind all available Vinculin in force-free environments such as at mitochondria^54^. This binding occurs even in the presence of actomyosin inhibitor, Y27632, and actin polymerization inhibitor, cytochalasin D and at a similar turnover rate to the stable mitochondria BAK protein. Taken together, these findings suggest that active Vinculin has a Talin-binding site that activates Talin in a force-independent manner^54,55^. The presence of active Vinculin also compensates for the mutated expression of the Talin actin-binding site (ABS3) in maintaining cell adhesion and focal adhesion assembly; this demonstrates that active Vinculin drives adhesion assembly even when the force necessary to expose the Talin–Vinculin-binding site is negligible^55^.

The aforementioned force-dependent Talin–vinculin interactions reported in in vitro purified and physical models^56,52^, as well as in cells exposed to a range of substrate stiffness^26^, form a framework to begin to elucidate the rigidity sensing capacity of cells and the mechanism of cell adaptation to external forces via focal adhesion machinery. Given the contradictory finding of force-independent talin–vinculin interactions in the emulated force-free environment, it may be important to consider intrinsic stress (defined as stress generated within cells and in the absence of external forces, and referred to as pre-stress or residual stress in some mechanics contexts) ubiquitously present in suspended and adherent cells (e.g., on gel substrates or glass). This instrinsic stress is maintained by the cytoskeleton; regardless of the cell adhesion state, the cytoskeleton would still allow talin–vinculin interaction in a purely biochemical manner provided that the right binding environment, such as phosphoinositide-rich membrane^57^, is available to recruit and to maintain actin and microtubule organization as required for a cell to remain viable.

### Formation of fibrillar adhesions, actin stress fibers

Fibrillar adhesions grow out from the focal contact and develop due to low tension and movable interactions with fibronectin, which then drives the segregation of the focal contact and fibrillar adhesion components^49,58^ (Fig. 2). While focal contacts contain high levels of vinculin, paxillin and phosphotyrosine (pTyr), fibrillar adhesions lacks these elements. In contrast to focal contacts, fibrillar adhesions are enriched in tensin and α_5_β_1_ integrin^49,59^. Fibrillar adhesions mediate fibronectin fibril formation and associate with thin actin that translocates centripetally from the focal adhesion^59^.

**Fig. 3:**
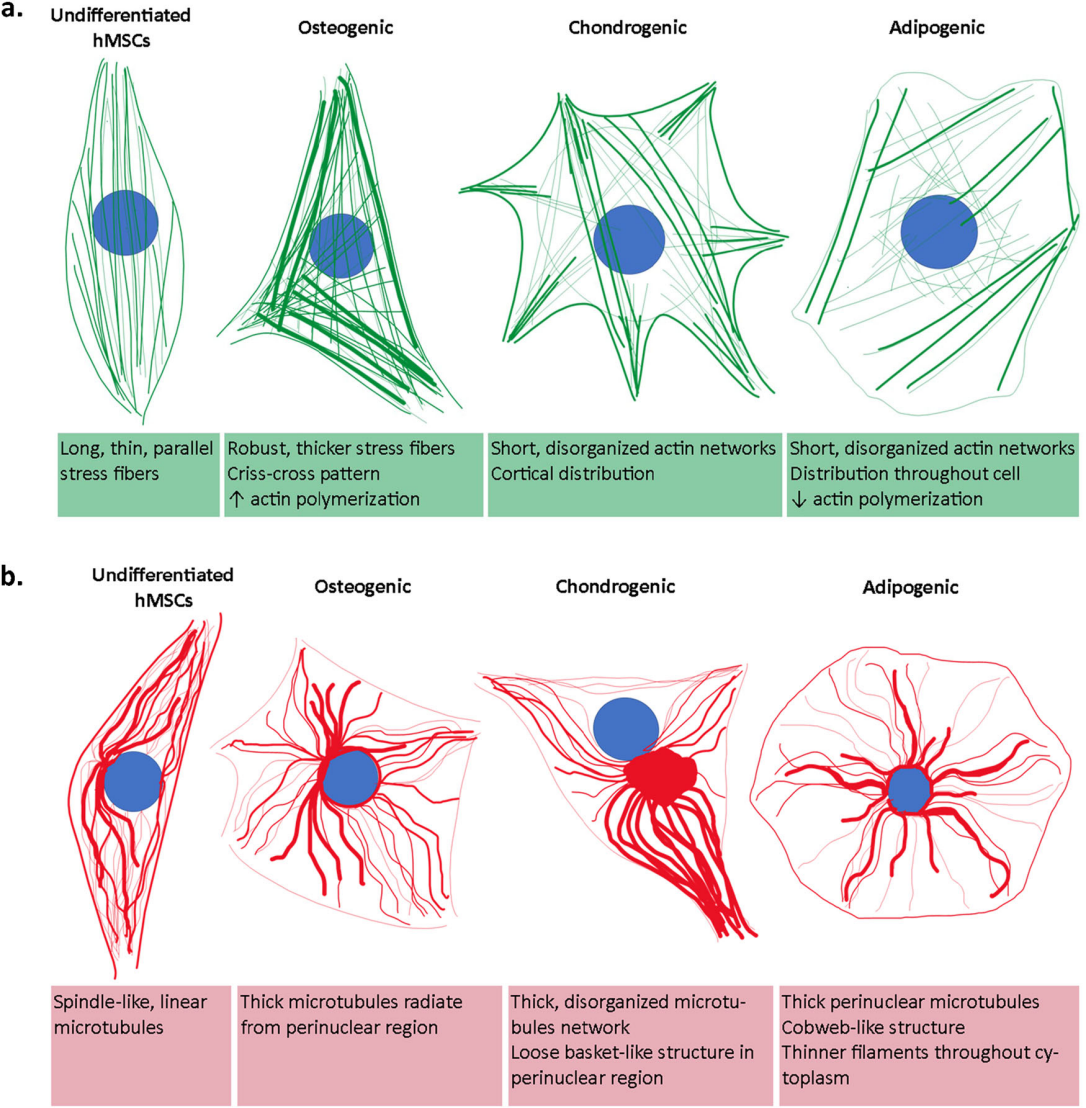
The unique remodeling of the cytoskeleton represents the adaptiveness of stem cells to their environment, shaping their differentiation responses. Similar to tracking of genetic markers that provide a fingerprint in time for processes of stem cell differentiation^5,39^, remodeling of actin (green) (**a**) and tubulin (red) cytoskeleton (**b**) correlates to incipient differentiation of mesenchymal stem cells (hMSCs). Regulation of actin and tubulin de-/polymerization results in specific spatiotemporal patterns of their fibers (shape, size, distribution, organization), reflecting the cells’ adaptation of structure and function to their prevailing mechanical and biochemical milieux^4^ (refs. ^116; 130; 202; 203; 204; 19^; and ^117^). In turn, changes in gene regulation and cytoskeletal remodeling scale up to create spatiotemporal patterns of tissues with specific architectures and functions^205^.

Similar to the dynamics of focal contacts, fibrillar adhesions also adapt variably to a range of substrate stiffnesses. For example, on soft substrates (0.8 kPa), fibrillar adhesions of HeLa cells are short and dot-like, whereas on stiff substrates (60 kPa), α_5_β_1_ integrin adhesion grows to a maximum length^60^. Gradual increases in adhesion length are Tensin1-dependent and can be observed with substrate stiffness gradients of 1–7 kPa; they plateau above 22 kPa^60^. This adaptive, and reversible clutch dis-/engagement, in response to stiff or soft substrates, as well as the interchangeable state of focal contacts and fibrillar adhesions, mechanically couple actin remodeling into thick stress fibers (SFs) or thin cables (Fig. 3).

Due to their strong anchorage by focal adhesions, actin SFs are characterized to be naturally under isometric tension, and they rarely shorten^61^. Local mechanical properties determine the strength of actin–integrin linkages and influence actin conformation and interaction with actin-binding proteins (ABPs) to regulate SFs dynamics^62^. Tension from rigid substrates induces SF localization, recruits polymerization factors profilin and α-actinin (Fig. 2d–f), and provides templates for focal adhesion maturation^63^. Investigation of yeast formin Bni1p-mediated actin polymerization shows that, in the presence of profilin, tension enhances the rate of formin-bound actin polymerization; in the absence of profilin the rate of formin-bound actin polymerization slows down^64^. A tension-dependent gating mechanism opens and closes formin’s conformation, and thus controls actin polymerization as required^65^. Under increasing tension up to circa 0.3 pN, the formin homology 2 (FH2) domain-bound actin barbed-end shifts from opened to closed conformation, which linearly decreases the rate of actin filament elongation^64^; however, this process is reversed by profilin-bound actin associated with the formin homology 1 (FH1) domain that dramatically increases the polymerization rate of (lengthening) actin monomers^64^. When endothelial cells are 10% stretched, binding of actin depolymerization factor, cofilin, is prevented due to reduction in the filament torsional amplitude or helical pitch^66^; this results in actin elongation. Cofilin binding contributes resistance to tension perpendicular to the direction of stretching by promoting actin turnover and stability of the actomyosin ring at the adherent junction^67^.

Thus, actin structural changes, and the associated impacts on affinity of ABPs and focal adhesion complex maturation (Fig. 2), present a multifaceted biophysical feedback loop, that when modulated mechanically or biochemically, may provide molecular mechanisms key to cytoskeletal remodeling and associated cellular structural and functional adaptation^4^.

### Microtubule remodeling

Microtubules, the stiffest cytoskeletal filaments (measured based on persistent length, ℓp,-dependent stiffness of in vitro reconstituted filaments where microtubule ℓp is >1 μm and actin ℓp is ∼17.7 μm^68^), function as compressive-resisting elements of the cell. Like a stiff rod that bends under compression, isolated microtubules bend into long-wavelength shapes (Euler buckling)^16^. However, within the gel-like cytoplasm, microtubules buckle into short-wavelength shape due to their natural lowest-energy bending state. The microtubules’ shorter buckling wavelengths in the mechanically coupled cytoplasm network demonstrate their capacity to bear large compressive loads from within and outside the cell (such as compression from adjacent cells), which influences cell shape^16^. This compressive bearing capacity is driven by microtubule polymerization at the cell periphery, which generates pushing or opposing force at the interface of the cell surface with its surrounding environment^16^. The flexural rigidity of the isolated microtubule, measured as buckling under compressive force, increases with increasing microtubule length but can also vary, when tubulin dimer subunits of the longitudinal filament of the same length are stabilized by different tubulin-modulating agents^69,70^. Stabilization of purified mictotubules with microtubules-associated proteins (MAPs), e.g., Tau, or Paclitaxel, differentially strengthens the bonds between the dimers, longitudinally and/or laterally; experiments stabilizing microtubules with Tau showed improvement of microtubules’ longitudinal (flexural) rigidity but not their lateral rigidity, highlighting the anisotropic nature of microtubule^71^. Interestingly, with rigidity directly proportional to length, the longitudinal force needed for microtubule buckling remains constant as microtubules grow longer, demonstrating microtubules’ higher resistance to longitudinal compared to lateral compressive forces^70^.

Microtubules actuate tension to facilitate pulling of motor proteins (dynein and kinesin) and chromosomes during cell division, particularly through a linkage at a specialized structure called the kinetochore. When purified complexes between the chromosome and the plus end microtubules are allowed to attach and are incubated in glycerol buffer to induce depolymerization, the mean length between tubulin and the kinetochore shorten over time and depolymerization occurs at the kinetochore, indicating that microtubule depolymerization provides energy for the chromosome’s poleward movement^72^. On 2D elastic media, kinesin regulates changes in wavelength and amplitude of microtubule buckling during exposure to compressive stress, enabling determination of critical strain at microtubule deformation^73^. These studies demonstrate that kinesin modulates mechanical properties of microtubules in response to changing substrate elasticity; analogous to actin, the continuous buckling enables microtubules to adapt to a wide range of elasticities.

The polymerization and depolymerization of actin and microtubules drive all intracellular processes, such as protein transport. The polymerization and depolymerization of actin and microtubules control force generation by the cell, as modeled and validated experimentally as a “Brownian Ratchet”—where chemical reactions during polymerization create protrusive forces by the cytoskeleton^74^. Using the optical trap method, the Dam1 (the load-bearing component of the kinetochore in yeast) coated bead anchors one end of purified microtubule moves the filament upon applied tension, and promotes the growth of microtubule protofilament in vitro^75^, proving that these physiologically relevant forces are achieved through microtubule polymerization and depolymerization. The mechanical role of microtubules is extensively reviewed in ref. ^76^, whereby polymerization creates a pushing force as the other end of the microtubule encounters a barrier (e.g., cell membrane), in contrast, depolymerization creates a pulling force for the other end attached to an object (e.g., kinetochore during division)^76^. The model of pushing and pulling forces demonstrate microtubule growth under tension as seen in mitotic spindle growth that pushes the centrosome or microtubule organizing centers (MTOCs) away towards opposite poles during cytokinesis.

### Intermediate filaments (IFs)

Intermediate filaments (IFs) are also involved in force generation and transmission, to and within the cell, during tissue development^77^. Within the large IF protein family, vimentin and lamin are widely known for their role in protecting cell and nuclear shape and integrity from the large deformations experienced during migration through 3D matrix typical of the EMT^13,78^. Exposure of endothelial cells to fluid flow induces heterogenous displacement of vimentin at the subcellular scale that continues with directional displacement in subsequent flow interval^79^, suggesting that vimentin redistribution upon shear stress may couple forces at the cell surface and nucleus and thus influence gene expression^79^. In migrating human foreskin fibroblasts (hFFs), vimentin fibers are found to align with actin branches in the direction of traction stress that determines cell directionality^80^. Vimentin’s colocalization with actin demonstrates its capacity to direct actomyosin contractile force orientation and homogenous distribution throughout the cell for energy conservation and an improved cellular load-bearing capacity^80^. Vimentin knockout cells exhibit significantly lower viability and lower cytoplasmic strength upon exposure to a large degree (up to 300%) of stretch^81^. Spatial correlation of vimentin and microtubules growing end protein (end-binding protein, EB1) trajectory reveals that vimentin provides templates for guiding microtubule polymerization and maintaining cell polarity during migration^15^. These studies give insight with respect to the role of IFs as intermediary structures between actin and microtubules, which facilitate force transmission and spatial organization, respectively, for persistent cell polarity in migration.

### Cooperative remodeling of the cytoskeleton

The mechanical environment of the cell includes both the prevailing endogenous mechanical stresses of dynamic physiological systems as well as the mechanical cues intrinsic to mismatches in stiffness at interfaces between the cell and its local environment. A series of studies using C3H/10T1/2 murine embryonic fibroblasts with mesenchymal multipotency reported cytoskeleton adaptation to volume and shape-changing stresses, incurred via increasing cell seeding density and/or exposure to fluid flow, which delivers a combination of shear and normal stresses to cell surfaces^5,7,21,82^. Cytoskeletal adaptation was observed as significant changes in the spatial distribution of actin and microtubules as well as in the cells’ mechanical properties that, together, correlated with changes over baseline in the gene expression of mesenchymal condensation markers^5,6,82^. Microtubule (measured as tubulin) concentration changed more significantly at a lower seeding density and with increasing distance from the substrate (basal to apical gradient)^6^. In contrast, changes in actin distribution were reported as more subtle across a range of stress magnitudes, with significant differences observed at higher cell seeding density^6^. This emergent anisotropy of cytoskeletal architecture highlights the distinct mechanical roles of actin and microtubules as well as their synergistic crosstalk at the subcellular scale, which acts to maintain stable force balances at the cell and tissue length scales. Specifically, tension-resisting actin tends to reorganize and thicken depending on the direction and magnitude of force, and compression-resisting tubulin buckles and bundles with respect to the substrate or proximity of neighboring cells.

Actin reorganization in integrin-mediated rigidity sensing is well-characterized. While an increase in rigidity enhances actin polymerization, traction force transmission onto the substrate, and promotes focal adhesion maturation, the role of microtubules in controlling structural adaptation has recently been shown to occur at the post-translational level. Studies of rat embryonic astrocytes cultured on substrates with increasing rigidity (1–48 kPa) report an increase in microtubule acetylation by α-tubulin acetyltransferase (αTAT1), a tubulin post-translational modification (PTM)^83^, which indicates the presence of long-lived, stable microtubules. The triggered mechanotransduction pathways involve αTAT1 recruitment to focal adhesion and interaction with Talin, which is abolished upon treatment with Y27632 (ROCK and actin polymerization inhibitor)^83^. In turn, microtubule acetylation is necessary for collective cell migration and actomyosin contractility as αTAT1-depleted cells show a lack of extended SFs, myosin light-chain phosphorylation (pMLC) and focal adhesion at the cells’ leading edge^83^. αTAT1 gene is also shown to carry several myocardin-related transcription factor (MRTF)-responsive elements, demonstrating that microtubule acetylation is required for actin polymerization^84^. Expression of αTAT1 mRNA diminishes when formin INF2 is silenced, and is accompanied by a repressed MRTF function, hallmark for high G-actin monomer and F-actin filament ratio^84^. As a whole, cells require the synergistic work of the entire cytoskeletal machinery to interact and mediate each other’s emergent responses, resulting in a greater force bearing, generating, and transduction capacity, with the putative minimization of energy expenditure during mechanoadaptation^4^.

Modulation of cytoskeleton de-/polymerization via the introduction of cytoskeletal modulating agents enables deciphering of cytoskeletal proteins contributions to mechanotransduction and mechanoadaptation. Through its dynamics, actin dominantly regulates cell strain-induced deformation, where upon inhibition of actin polymerization by latrunculin A, deformation increases significantly even at small strain^85^ Stabilization and induction of polymerization by Jasplakinolide compensates for this deformation, producing a reinforcement response through actin lengthening. Based on literature reports, dynamic reinforcement and retraction help actin adapt to strain in three-dimensional (3D) engineered tissue construct built from chicken embryonic fibroblasts; when exposed to small strain over 30 min, reinforcement occurs through increase in SFs’ polymerization, thickness, and alignment, and protrusion in the direction of stretch^86^. At higher strains, retraction occurs for all SF orientations through an increase in SF depolymerization^86^. At the tissue scale, in 3D under gradually increasing strain, actin depolymerizes and becomes more elastic as shown with decreasing tissue storage stiffness and pre-stress, contributing to a tissue strain softening response^87^. Upon strain removal, actin polymerization increases as a measure of recovery rate that is proportional to the depolymerization rate in a given strain duration^87^. Microtubules do not contribute to the strain softening behavior however, inducing microtubule depolymerization with Nocodazole increases tissue pre-stress and thus increases tension recovery^87^. In two-dimensional (2D) culture, stabilization of actin with introduction of small strain result in ∼50% increase in cell deformation, while interestingly, at large strain, results in an insignificant cell deformation^85^. Correspondingly, chemical agent-induced microtubule stabilization, i.e., by Paclitaxel (known as the drug *Taxol*), results in concentration-dependent cell deformation at large strain, where at concentration 25–100 nM relative cell deformation increases but above 100 nM decreases^85^. This shows that actin and microtubule cooperatively maintain cell mechanical integrity, which at larger strain, is regulated predominantly by microtubules^85^. In addition, there is a clear time and dose-dependent response of cytoskeleton to local mechanical stretch and chemical inhibitors which impart cell/tissue-wide changes. This dose-dependent chemical modulation of actin and microtubule could link the cell stress and deformation threshold which itself may be specific to cell type and mechanoadaptation capacity. Future work on the development of nanomaterials for controlled delivery of those chemical modulators and region-specific targeting of actin and microtubules would provide a valuable tool to achieve specific cytoskeleton structure and function, for targeted tissue healing and regeneration.

## Cytoskeletal adaptation in development and healing

In both prenatal development and postnatal healing, architectures emerge during tissue neogenesis, resulting in creation of appropriate structure and function, as manifested in cell phenotype, e.g., cell morphology, lineage, and generation of ECM. Cytoskeleton reorganization plays a role in the process of achieving mechanical homeostasis in tissues, i.e. the balance between tissue growth and degradation at steady state, which represents the architectural adaptation of cells over time concomitant to the facilitation of biological processes like cell division, migration and differentiation in a most energy efficient manner^4^. Understanding how stem cells achieve equilibrium through mechanoadaptation, together with their energy adaptation (the energy balance required to induce changes in the cytoskeleton, synthesize or degrade materials), will help to predict stem cell behavior and lineage commitment in context of multi-time- and length-scale physiology of an organism^4^.

Cytoskeleton filament descriptors (e.g., length, orientation, thickness, spatial distributions) provide quantitative indicators of cellular adaptation and differentiation across time and space, during tissue development and healing. The elucidation of the mechanisms underpinning stem cell mechanoadaptation will also help to decipher how the cytoskeleton remodels to intrinsic forces of development and healing. During morphogenesis, wound healing or tumor progression, cell adaptation to the ever-changing tissue mechanical properties (e.g., rigidity) relies on cytoskeletal and cell adhesion machinery. Rigidity sensing involves the interdependence of cell-ECM (integrin) and cell–cell (cadherins) adhesion that influences traction forces generated by actin and force transmission to the ECM and neighboring cells toward achieving tissue tensional equilibrium^88^. Different types of cells, such as fibroblasts and epithelial cells, spread on stiff substrates or merge as tissue aggregates on soft substrates over time, which demonstrates the differential adhesion and myosin II-dependent contractility that regulates the tissue-building response^88^. In zebrafish gastrulation, cadherin-mediated cell adhesions provide a mechanical template for cortical tension development, in which to control cell contact expansion during cell sorting^89^. Cortical tension controls contact expansion that determines the anchoring strength of actin to E-cadherins in individual cells, which again is in concert with actin polymerization^89^. The polymerization state of actin represents cell adaptation to the differential surface tension required for tissue compaction or folding. Cadherin-mediated adhesion between cells in the multicellular embryo allows cells to initiate contact expansion and to sense the accumulated tension as a means to control further contact expansion and to efficiently sort cells, i.e. cell patterning.

All tissues are built upon two architectural templates referred to as epithelial (sheet-like) and mesenchymal (globular)^1,4^. During early development, the interconversion between epithelial and mesenchymal states (epithelial-to-mesenchymal transition, EMT ↔ mesenchymal to epithelial transition, MET) is mechanically driven—involving a gradient in surface tension which is highest at the boundary of ingressing and non-ingressing cells (Fig. 1b) and requires high actomyosin accumulation and contractility^89,90^. Cells undergoing EMT generate apicobasal forces with myosin II accumulation, the forces that induce tissue invagination^91^. The mechanical control of cell migration during tissue morphogenesis appears more dominant than the control by chemical cues. As shown in in vivo *Xenopus laevis* embryo, neural crest collective cell migration or EMT depends on the stiffening of the adjacent mesoderm (known as durotaxis) which occurs as mesoderm increases in cell density in the progression to gastrulation^48^. Inhibition of myosin in the mesoderm decreases its stiffness and consequently blocks neural crest cell migration^48^. In another in vivo study, while neural crest cells seem to collectively migrate upon sensing the chemoattractant released by the underlying placodes (chemotaxis), the neural crest cells chase the placodes that concurrently ‘run away’ creating a stiffness gradient governing neural crest migration^46^. This interaction between neural crest cells and the placode is mechanically driven and the loss of stiffness gradient due to the ablation of placodes diminishes neural crest cells migration^46^. With controlled stiffness gradient, neural crest cell clusters establish polarized actomyosin contraction that predicts their direction in relation to the matrix stiffness along with polarized vinculin focal adhesion distribution^46^.

At the earlier stage of 8–16 cells in the mouse embryo, the accumulation of Myosin II around constricting cells drives the allocation of the first inner cells and that myosin II distribute heterogeneously and correlates with difference in contact angles between the cells^90^. The loosening of epithelial cell attachment allows for apical constriction that is regulated, respectively positively and negatively, by *twist* and *snail* mechanosensitive transcription factors^92^. Stress fibers (SFs) continuously cycle and are heterogeneously distributed, with an accumulation of actomyosin bridges between cells, that creates differential tension and thus drives cell ingression^93^. A tension gradient dictates the spatial pattern of activation and the nuclear translocation of MRTF-A, that couples Rho-GTPase activation; Rho-GTPase in turn facilitates the addition of G-actin monomers to enhance actin polymerization^94,95^ (Fig. 2f). Thus, during EMT, the thin cortical actin of epithelial cells remodels into thicker parallel stress fibers, as cells acquire migratory features following rapid formation of lamellipodia at the leading edge of the readily migrating cell^96^ (Fig. 1b). During axis formation in the amniote embryo, coordinated tissue elongation is required in multiple regions^97^ and actin filopodia, serving as antennae for cells, exert traction force to drive cell convergence and extension movement along the anteroposterior (AP) axis^98^. In a zebrafish embryo model, actin filopodia are shown to carry Wnt8a, a Wnt signaling protein essential for development, towards their distal tips in cell extensions during AP patterning. Region-specific folding in the ventral-lateral axis shows that cells within six rows away from the axis vary in apical area, which reflects the requirements for a gradient of apical constriction, and thus a gradient of actomyosin contractility across the developing embryo structure^98^.

A similar process repeats in postnatal growth and healing where the cytoskeleton remodels and redistributes to accommodate the changing state of tension in the tissue. In wound healing studies, laser ablation-induced wounding of a HeLa cell monolayer^99^ results in uniform F-actin enrichment, increasing with time. Rapid wound closure relies on heterogeneous actin and myosin distribution and contractility forces around the wound^100^. Supracellular actomyosin cables around the wound promote collective cell migration and direct traction force towards the wound through their staggered contractility^100,101^. In a chick amnion wound model, an actin cable or purse-string contraction results from existing cortical actin rearrangement within the cells, which appears within 10 min and is Rho-dependent^95,102^. However, in mature tissue, wound closure predominantly involves cell crawling, which is driven by actin dynamics and regulated by complex biochemical signaling (e.g., Rac-GTPase-dependent lamellipodium protrusions)^103,104^. Commonly studied in a Madin–Darby canine kidney (MDCK) cell monolayer, wound closure in mature tissues appears to be facilitated by high protrusive activity of cells at the margin and even few hundred microns behind the edge of the wounds^103^. Up to 15 rows of cells behind the wound edge extend their lamellipodia underneath the basal region of cells ahead of them, and this movement occurs at the rate inversely proportional to the distance from wound margin^103^.

As mathematically modeled in ref. ^104^, the mechanical process of wound closure by cell crawling is enhanced by cell–cell adhesion; cells at the wound margin exert pulling force to the cells behind them into the wounded region, while in regions further from the wound, the force distribution causes instabilities and vortices, causing the cell monolayer to behave collectively as viscoelastic material^105^. The model indicates that wound closure is a mechanically driven process that can be modulated by biochemical signaling^104^. Using micropillar removal method, the influence of different size and shape of the wound on wound closure mechanisms can be studied—depending on the size of wound purse-string contraction and cell crawling contribute differently in MDCK monolayer gap closure, where closure of large gaps greater than 150 μm in diameter is dominated by lamellipodial extension where closure time increases with Rac inhibition^106^. In contrast to the classic scratch assay that causes cell damage, lamellipodium protrusion or lamellipodial-driven crawling dominates closure of small gaps less than 20 μm in diameter instead of the purse-string mechanism; which is likely initiated by cell damage associated apoptotic signaling^107^. In this case, the small gap closure time is independent of Rac inhibition, proving that wound gap size influences lamellipodium protrusion-governed closure kinetics—a mechanism that is independent of Rac and Rho, and is purely physical (e.g., density-dependent)^106^. Wound geometry (curvature and shape) also influences the closure behavior, that with a square or ellipsoidal wound shape of the same MDCK monolayer, lamellipodial activity is favored in regions of low curvature and contributes to faster closure time compared to more circular wounds^106^.

Actin serves as a mechanical sensor (also known as a “mechanostat”) and adapts by sensing and generating forces that underpin the mechanobiological feedback stabilizing forces between cells and matrix and overall tissue structure^108^. As shown in C2C12 myoblasts exposed to mechanical stretch and elongated geometry, actin plays a key role in preserving viability of myoblasts much more than other cell types (e.g., endothelial or fibroblasts) exposed to the same cues^109^. Exposure to Cytochalasin D releases actin tension in myoblasts confined to elongated geometries and further rescues cell viability^109^, proving that myoblast actin serves as a prerequisite for single cells to function under mechanical stretch even before they build muscle tissues. How actin redistributes and facilitates specific cells to perform their function in context of their mechanical environment is not yet understood.

In development, microtubules are required for epithelial folding, to generate a pushing force to the apical surface while stabilizing the actomyosin ring in the adherent junction^76^. During the neurulation stage of development, the transition from the neural plate to the neural rod, concomitant with cell elongation along the dorsal midline, demonstrates reorganization of microtubules from radially arranged and evenly distributed throughout cytoplasm to long linear bundles aligned along the future apical–basal surface^110^. The resulting higher ratio of detyrosinated tubulin (stable microtubules) to the total tubulin suggests that microtubules acquire more stability as neurulation progresses, and this stability is controlled by MAPs. The gradient of MAP1b expression across neurulation stages is consistent with the distribution of stable microtubules, which also indicates higher transport activity of ABPs and crosstalk with actin to facilitate filopodia protrusions for directional migration^110^. In a *Xenopus laevis* embryo wound, microtubules reorient to a radial structure and form thicker bundles in the cell cortices within 10 min of wounding^111^. Microtubules are also required in the accumulation of E-cadherin at cell–cell contact and assembly of actomyosin at this E-cadherin-rich junction^112^, as microtubule dynamics controls E-cadherin distribution in filopodia that facilitates junctional rearrangement for actomyosin cable localization during wound healing^113,114^.

The emergent remodeling of the actomyosin and microtubule network and their crosstalk reflect the complexity of developmental cues across length scales and the capacity of stem cells to adapt and achieve structural stability in newly formed tissue. With an increased understanding of the mechanical implications of cytoskeleton remodeling for tissue development and healing, the next challenge is to test ways to emulate mechanical cues relevant to cytoskeletal adaptation and to elucidate how cells and their cytoskeletons achieve force balance to promote tissue healing.

## Cytoskeleton remodeling for targeted tissue neogenesis

Ultimately, tissue engineering and regenerative medicine aim to control stem cell differentiation to regenerate tissue inhabited by cells of desired lineages, which create appropriate extracellular matrix, arranged in desired architectures to optimize function in the prevailing physiological environment. Cell structural parameters, such as spreading, diameter, orientation, and density, are modulated by cytoskeletal dynamics, and conversely cytoskeleton rearrangement dictates cell structure, creating a feedback loop that manifest specific tissue neogenesis^106^. Tracking of cytoskeletal dynamics helps to identify early lineage divergence of stem cells and provides a valuable tool to identify relevant cells among a heterogenous stem cell population^115^. The use of cytoskeletal modulating agents to drive differentiation started over two decades ago, but the possibility to engineer the cytoskeleton to achieve targeted differentiation states remains a gap in the field.

### Mechanically and biochemically induced differentiation

The structural remodeling of actin in mesenchymal stem cells exhibits distinct hallmarks for identification of unique differentiation pathways (Fig. 3). Structural changes in actin occur within 14 days of osteogenic differentiation achieved using induction medium; these changes include thicker and more disordered SFs with criss-cross patterns (Fig. 3a), compared to the relatively unidirectional, parallel thin fibers observed in undifferentiated control cells^116^. In contrast, during chondrogenic differentiation achieved using induction medium, SFs gradually disappear and peripheral actin arches become less defined^117^. On day 14 of chondrogenic induction, the alignment of the actin subdomain is no longer unidirectional but rather is organized into networks, and by day 21, thinner cortical actin is present as cells elongate^117^. Across mesenchymal stem cell differentiation pathways, microtubules appear to maintain a similar radial structure, emanating from the centrosome around the perinuclear region (Fig. 3b). Cell shape changes are likely to be mediated by actin. Per example, the cuboidal shape of osteogenic-differentiated cells is due to enhanced actin polymerization and thicker SFs in the cell periphery. The rounded, adipogenic-differentiated cells reflect the decreased actin polymerization throughout the cytoplasm. Taken as a whole, a consensus formed through a plethora of published studies notes the negligible role of the changing global structure of microtubules in facilitating shape changes during differentiation^118,119^. However, the regulatory role of microtubules during differentiation is more evident in the upstream molecular interactions, controlling nuclear deformations, gene expression, and other cytoskeleton filament growth^83^.

Microtubules modulate nuclear shape through their close association with lamins and nesprin nuclear envelope proteins (IF protein family), whereby disruption of the microtubules’ dynamic de-/polymerization impacts expression of those proteins and adipogenic differentiation^19^. Microtubule pulling forces control nuclear deformation during hematopoietic stem and progenitor cells’ (HPSCs) myeloid differentiation^120^. The modulation of cytoskeletal tension by confining cells to different geometries, such as a flower or star pattern, respectively, prompts differentiation towards either adipogenic or osteogenic fate^121^. Differences in subcellular curvature across these shapes influences the adhesion points and associated cytoskeletal organization, which consequently directs downstream lineage progression. Inhibition of microtubule polymerization with Nocodazole (an anti-mitotic agent targeting microtubule assembly) results in strong osteogenic preference in both flower and star patterns, circumventing the influence of shape on cell differentiation^121^. Reduction in microtubule polymerization enhances actin contractility in both patterns that favor osteoblastic shape. In contrast, treatment with Cytochalasin D (inhibitor of F-actin assembly) impedes SF formation, driving adipogenic differentiation irrespective of geometry. This shows that although directed differentiation (structure–function acquisition) requires both coordinated actin and microtubules cues, yet the role of microtubules is more relevant at the molecular level and visible structural changes brought by actin will not be possible without molecule transport performed by microtubules.

Throughout 21 days of chondrogenic differentiation induction, non-radial arrays of microtubules gradually increase, with the presence of disorganized, highly condensed non-centrosomal microtubules at day 7, and basket-like microtubule bundles around the nuclei^117^. After 1 day in adipogenic induction medium, microtubules appear elongated and evenly distributed across the cytoplasm. Over 14 days, microtubules remodel to adapt from a fibroblastic to a rounded adipocyte morphology resulting in increased tubulin density, a higher degree of buckling in cell periphery and loosely distributed fibers in the cytoplasm to accommodate the formation of lipid droplets^19^. During adipogenesis, microtubule remodeling depends on α-tubulin acetylation (tubulin post-translational modification) which promotes conformational change and severing of microtubules (breaking microtubules along their length) to facilitate acquisition of adipocyte morphology^122^. Cytoskeleton reorganization thus serves as an indicator of unfolding lineage commitment where the temporal transition of filament remodeling shows the stage of differentiation.

Across length scales, cytoskeleton remodeling serves as both a physical as well as an emergent indicator of differentiation and/or specific tissue neogenesis in response to both mechanical as well as biochemical changes in the cells’ environment. Myriad experimental studies demonstrate the profound impact of mechanical loading on cytoskeleton remodeling. In this sense, the cytoskeleton is a sensitive sensor, actuator and transducer that responds to mechanical signals as diverse as loading direction, magnitude, duration, and frequency *etc*.^4,33^ These mechanical loading effects, transduced from the organism (meso) to the molecular (nano) scale, i.e. top-down, trigger a sequence of molecular events that result in tissue and organ scale remodeling, i.e. bottom-up^2^. For example, three-point bending of the murine tibia induces tension on the medial side of the mid-shaft, opposite to where the direct point load is applied^17^. In response, cells residing in the periosteum (covering all bone surfaces except where cartilage is present) exhibit both a disorganization of actin networks and a reduction in actin bundles^17^. Subsequent application of the same load in long-pulses (39.5 s load and 0.5 seconds rest) restores actin SFs in the periosteal cells within four days of loading, with a higher density around the nuclei, and thinner SFs evenly distributed throughout the cell^17^. Concomitant to this increased cytoskeletal remodeling, alkaline phosphatase (ALP) activity increases, and periosteum hypertrophy and woven bone formation are observed. Hence, after 14 days of long-pulse three-point bending exposure, significant bone formation is observed at the organ (meso) scale. Similar effects are observed upon treatment of periosteal tissue with Cytochalasin D, an actin polymerization inhibitor, which induces actin disorganization. The reduction in actin SFs intensity resulting from cytochalasin D treatment mimics that induced by mechanical loading, with concomitant periosteal hypertrophy and increased periosteal thickness^17^.

Not only transduction of macroscale mechanical loading cues (e.g., running exercise generates 1500–3500  με strain in bone^123^) but also biophysical stimuli associated with high-frequency, low-intensity vibration (LIV) (up to 45 Hz–0.6 g generates 30 με strain in bone matrix of 8-week model^124^) are readily transduced to and sensed by cells, in turn activating cytoskeletal structural remodeling for musculoskeletal function across length scales^18^. During osteogenic differentiation induced by exposure to high-frequency (100 Hz–0.125 g) LIV and induction medium, the longitudinal axis of actin SFs predominantly align with the primary axis of vibration orientation^18^. This increased SF alignment also contributes to increased cell stiffness, measured via atomic force microscopy (AFM)^18^. As a whole, these data suggest that biophysical forces aligning and directing actin polymerization in a predominant direction contribute to tensile stress and stiffness in cells, which has been shown experimentally to support osteogenesis. Similarly, the application of cyclic hydrostatic pressure (CHP) on C3H/10T1/2 murine embryonic fibroblasts enhances actin expression, imposes the breakdown and centripetal translocation of intermediate filaments (IF) toward the perinuclear region, which induces osteogenic differentiation^20^. Treatment with IF-disrupting agent, Withaferin (WA), mimics the IF remodeling and differentiation resulting from CHP exposure, demonstrating that IF remodeling is necessary for CHP-induced osteogenic differentiation^20^. While increased actin polymerization has long been associated with osteogenesis^116^ comparatively little is known about the role of IF in mechanotransduction and cytoskeletal remodeling; given the colocalization of IF and actin suggests their interaction and crosstalk play a putative role in facilitating osteogenesis during CHP exposure. Historically, CHP exposure has been associated with chondrogenesis^125,126^ and further elucidation of the role of IF in modulating osteogenic versus chondrogenic differentiation responses of mesenchymal stem cells is warranted, given the potential relevance of IF and IF-actin interactions in directing endochondral versus intramembranous bone formation^126,127^.

Indeed, cytoskeletal remodeling is sensitive to the magnitude, direction, duration, and temporal sequence of the applied mechanical forces as well as to different lineage-specific biochemical cues (as summarized in Box 1). Hence, cytoskeleton remodeling is necessary for stem cell differentiation, although there is no unifying feature of the cytoskeleton that indicates differentiation preference (e.g., actin alignment and disorganization can occur during osteogenic differentiation stimulated with different cues). The increased rate of actin polymerization can generally serve as indicator for osteogenesis; however, for such remodeling to take effect on the SF thickness or alignment would greatly depend on the different time scales. While the radial array of microtubule seems notable in adipogenic or chondrogenic differentiating cells, microtubules’ role in mediating mechano-responses is more predominant in the signaling such as through its acetylation^83^. In addition, there is no clear trend in which type of mechanical stimulation can induce specific differentiation. Emulating endogenous dis-/reorganization of the cytoskeleton through exogenous treatment with cytoskeleton-disrupting agents will enable the elucidation of molecular mechanisms of stem cell mechanoadaptation. Such approaches may also be applied proactively to replicate differentiation responses of stem cells to mechanical modulation. As a whole, the previously described studies suggest that the pathways initiated from diverse biophysical and chemical cues intersect via cytoskeleton dynamics. The definition of protocols, precisely disrupting cytoskeleton dynamics, e.g., mechanically or chemically, to prompt a specific fate will only be possible through integrative approaches of controlled delivery of mechanical, biochemical and physical cues which enables mapping of unique cytoskeletal responses and cell fate. Approaches including the development of mechanically responsive and/or biochemically active materials and/or devices, nano to micro scale matrix engineering, drug delivery systems, imaging platforms, and computational modeling could contribute to further linking cytoskeletal adaptation and stem cell lineage commitment.

## Cytoskeleton-targeting agents to guide SC differentiation

SC differentiation upon modulation of actin and microtubule polymerization: As noted above, effects of cytoskeletal modulating agents (some of which are typically used in a medical context, e.g. chemotherapy drugs) can mimic cytoskeletal remodeling effects induced by biophysical stimuli. Over two decades ago, inhibition of actin polymerization with Cytochalasin D or Latrunculin B was reported to induce chondrogenic differentiation in chick embryo MSCs, through increased activation of a broad functioning signaling protein kinase C alpha (PKCα)^128^. Even earlier work revealed that Cytochalasin D promoted chondrogenic differentiation in chick limb bud culture^129^, which is consistent with the disappearance of actin SFs post-chondrogenic induction in bovine MSCs^117^. With regard to osteogenesis, however, Cytochalasin D treatment reduces ALP activity and osteocalcin expression^130^. In contrast, Cytochalasin D enhances adipogenic differentiation as shown by significant increase in oil red O (lipid-specific staining) and adipogenic gene marker, *Ppar-γ*, expression observed at 3 days post induction in human bone marrow-derived MSCs^130^.

The observation of the interplay between switching on/off actin polymerization and lineage commitment is further supported by the more recent findings that cells exhibit reduced osteogenesis and dose-dependent ALP activity in cells treated withCytochalasin D^131^. Stabilization of actin with phalloidin enhances cell viability and increases ALP activity^132^. Similar effects are observed upon knockdown of actin-depolymerizing factors, *Cofilin1* and *Destrin*^131^. Using the same experimental setup, upon *Cofillin1* and *Destrin* knockdown^133^, adipogenesis is enhanced by Cytochalasin D but reduced by phalloidin. Switching on/off microtubule polymerization via Nocodazole or Paclitaxel treatment further clarifies these agents’ antagonistic effects in adipogenic differentiation of human Adipose-derived Stem Cells (hASCs). As tubulin expression decreases and increases upon Nocodazole and Paclitaxel treatment, respectively, the opposite occurs for *Ppar-γ* expression; Nocodazole-treated cells express significantly higher *Ppar-γ* and the Paclitaxel treated cells expressed lower *Ppar-γ*^19^.

Taken together, these findings suggest that cells respond to both internal and external cues, where the structure state of actin or microtubules within the cell dictates their function in the prevailing environment over time, reaching a state of homeostasis as relatively stiffer osteocytes or a softer adipocytes, representing the two bounding extremes of MSC fates^4^. The polymerization states of actin or tubulin may serve as indicators of a differentiation state; although the decision for MSCs to pursue a particular fate is far more complex than switching on/off actin or microtubule disassembly using chemical agents, there is a clear trend where increasing actin or microtubule polymerization supports osteogenesis while inhibiting their polymerization supports adipogenesis. Conversely, if cell shape changes induced by mechanical and biochemical factors can be replicated by the controlled spatiotemporal delivery of these agents, then such delivery may be used to guide targeted SC differentiation. To this end, a reference library of actin- and tubulin-modulating agents (summarized in Boxs 2 and 3, respectively), together with the further delineation of differentiation pathways, is essential to decipher their complex interplay.

Cellular function and signaling pathways upon targeting actin and microtubule polymerization: Paclitaxel reduces the capacity for differentiation, proliferation, and migration in hASCs and hMSCs in a dose-dependent manner^134,135^. Interestingly, the introduction of surface topography at various length scales improves hMSCs’ resistance to Paclitaxel^136^. Namely, distinct engineered topographies modulate stem cell shape and membrane curvature, resulting in a higher order of transmembrane focal adhesions and an increase in actin polymerization at cell adhesion sites^29^, which improve cell survival, metabolic activity, and the putative capacity to reach homeostasis and lineage commitment^4^. Together, the increased resistance of hMSCs to Paclitaxel and the increased cellular adaptation to enforced shapes (surface curvature) may link underpinning mechanisms of actin and tubulin crosstalk by increasing cellular conformance and minimizing energy expenditure (metabolism). Hence, if either actin or tubulin function is inhibited due to physical, mechanical or chemical cues, then respectively tubulin or actin could compensate by responding dynamically to maintain cell baseline function. Without other mechanical means to stimulate them, cells would simply enter a non-proliferative state^137^ or cellular quiescence^138^ that would serve as the preferred “minimum power expended” state^4^ while also maximizing the cell’s differentiation potential at later timepoints.

Treatment of cells with microtubule polymerization inhibitors results in complex cytoskeletal responses. For example, in one study, microtubule polymerization inhibitors TN16, Colchicine, and Nocodazole were shown to increase BMP-2 promoter activity and bone formation, a modulation mediated through the hedgehog pathway^139^. In another study, treatment with actin polymerization inhibitors, Cytochalasin D and Latruculin A, and depolymerization inhibitor, Jasplakinolide was shown to enhance adipogenesis but to reduce osteogenesis in biochemically induced hMSCs^140^. The signaling proteins ERK (mitogen-activated protein kinase) and AKT (serine/threonine kinase) are central to events controlling cell proliferation, differentiation, and survival, and mechanical cues influence their activation. When mechanical stress is applied to integrins, both Cytochalasin D and Latrunculin A inhibit ERK and AKT activation, while Jasplakinolide exerts no such effect^140^. This indicates that actin stability is required in osteogenesis via the AKT pathway and is further enhanced with mechanical stresses presentation to integrins.

**Fig. 4:**
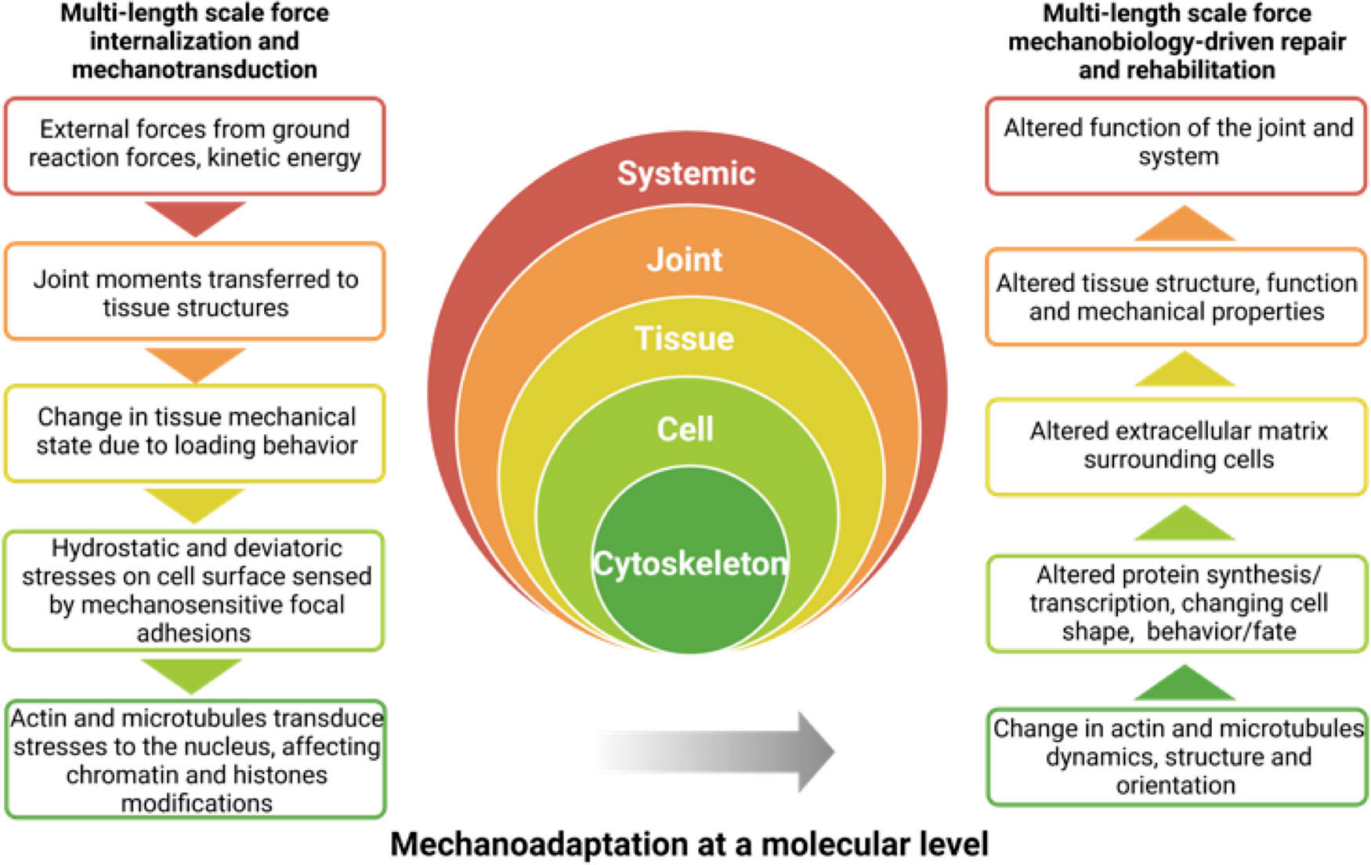
Multiscale mechanoadaptation as a rationale for design of engineering tissue templates, materials or devices for regenerative medicine and for physical therapy protocols in the context of cytoskeletal remodeling. Transfer of forces  experienced by an organism to its constituent organs, tissues, and cells (left, top to bottom) drives the multiscale structure–function adaptation of the cytoskeleton, cells, tissues and organs, to the dynamic mechanical environment of the organism, which is essential to healing (right, bottom to top). At the molecular level, focal adhesion complexes mediate outside-in signaling and mechanically link tissue rigidity to cytoskeleton dynamics, regulating cytoskeletal interactions with adapter proteins, and force transduction to the nucleus. The mechanical signals, transduced as actin and microtubule dynamics, determine cell shape and force generation, resulting in tension equilibrium at tissue and other length scales (adapted with permission from ref. ^2^).

The relationship between cytoskeletal remodeling and differentiation appears to be ubiquitous across cell types. Recently established links between the polymerization state of actin and stages of pancreatic differentiation in human pluripotent stem cells (hPSCs) point to new approaches; in this case, targeted modulation of cytoskeleton state and stem cell fate may pave a path for a novel cell-based diabetes therapy, where insulin-producing β-cells are needed^141^. During β-cell differentiation, treatment with Latrunculin A results in higher expression of *NEUROG3* (late pancreatic marker) but not *NKX6-1*^*+*^^141^ (early pancreatic marker), suggesting that actin depolymerization instead promotes glucagon secreting α-cells^142^. Five pancreatic progenitors of different stages of pancreatic development can be found within one cell population, demonstrating that their proportion varies with the actin and microtubule depolymerization stages following Latrunculin A and Nocodazole treatment, respectively. Actin depolymerization results in almost equal expression of the four pancreatic and endocrine progenitors, but lowest expression for exocrine progenitors. In contrast, microtubule depolymerization results in exocrine progenitor expression accounting for two-thirds of the total population expression^141^. Targeting the cell’s cytoskeleton polymerization state with respect to its differentiation stage thus strengthens the link between mechanical and biochemical regulation in controlling differentiation outcome. Fine-tuning of the treatment regimens and the mode of delivery to achieve specific phenotypes and cell behaviors for different stem cell types could provide new means to understand the process collectively and across length scales (Fig. 4).

Box 2 Modulation of actin cytoskeleton dynamics for targeted stem cell differentiation

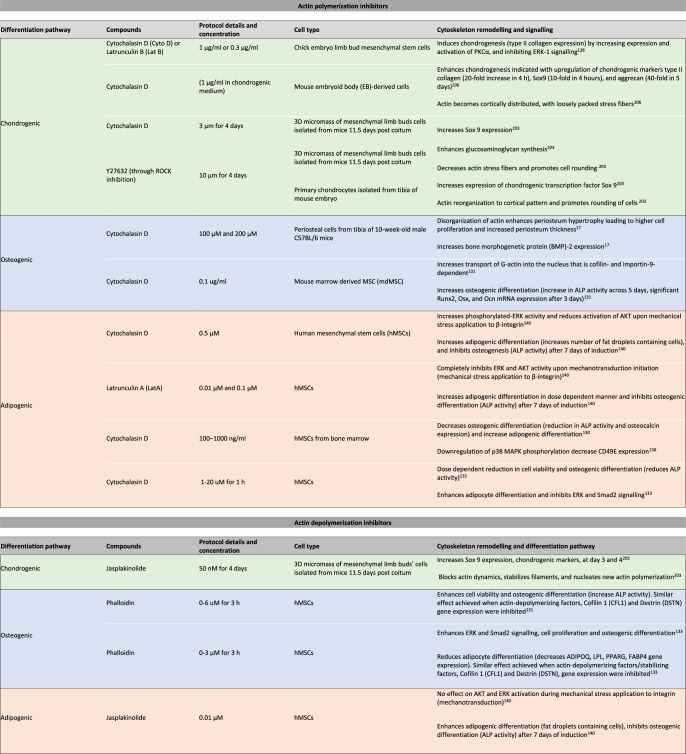

Box 3 Modulation of tubulin cytoskeleton dynamics and its implications for cellular function and differentiation

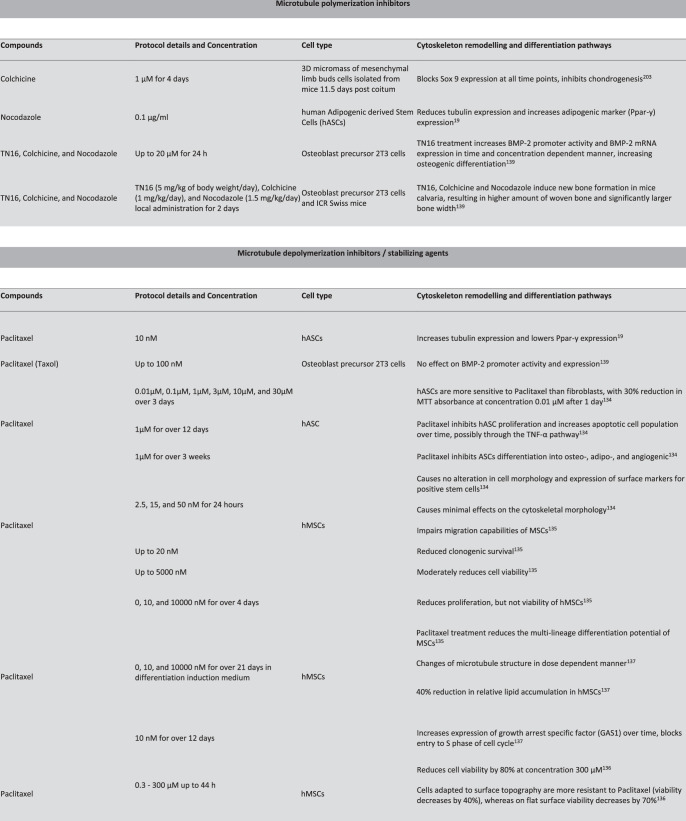

## Quantitative microscopy of cytoskeletal adaptation

**Fig. 5:**
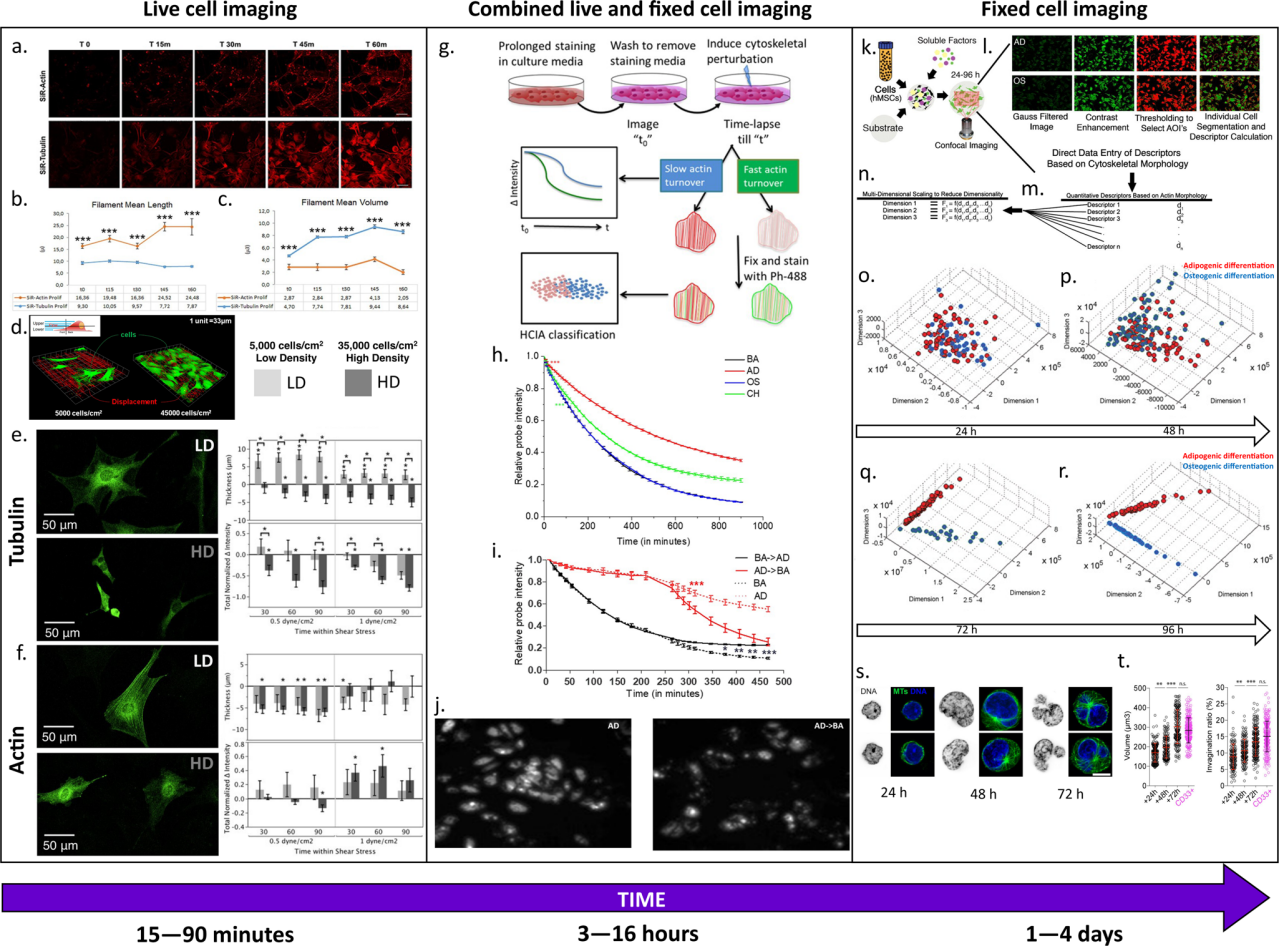
Quantitative imaging protocols for monitoring spatiotemporal changes in cytoskeletal structure during stem cell adaptation to cues driving proliferation and differentiation. Live cell, fixed cell, and combined imaging methods have proven to be useful in monitoring cytoskeletal changes indicative of lineage commitment as a function of time, with time resolution from within minutes to hours (live cell imaging), and or days, e.g. 24 h (fixed cell imaging). **a** Dynamic changes in actin and tubulin cytoskeleton (quantified as filament length (**b**) and volume (**c**)) are observed as early as 15 min during neural differentiation of induced pluripotent stem cells (iPSCs)-derived neurons via time-lapse imaging^144^. Simultaneous imaging and introduction of volume-changing stress (seeding density) and shape-changing stress (flow)^39,82^ (**d**) enable elucidation of spatiotemporal cytoskeletal adaptation to controlled mechanical cues. Spatial distribution of actin (**e**) and tubulin (**f**) are measured at 30 min intervals as fluorescence intensity within the thickness of the cell and the total cell volume^6^. **g** Combining live and fixed cell labeling and imaging of actin using fluorescent-conjugated SiR-actin (SA) and phalloidin, respectively, SiR-actin-based measurement of actin turnover (SMAT) analysis distinguishes MSC differentiation (**h**) toward adipogenic (red line), chondrogenic (green line), and osteogenic (blue line) lineages, at timepoints as early as 1 h. Reduction in probe intensity (**i**) is observable within a few hours of switching from adipogenic induction medium (AD) to basal medium (BA), as shown qualitatively (**j**)^115^. Actin cytoskeleton morphometric descriptors, including shapes, intensities, and spatial distribution, represent the apparent changes that are readily detected within 24 h of hMSC differentiation. **k** The lineage commitment propensity of hMSCs cultured in respective differentiation induction media on various substrates could be parsed using descriptor-based computational modeling. The resulting confocal images are processed using Gaussian filter, enhanced and segmented for each single cell (**l**) to generate 43 descriptors (**m**). **n** Multidimensional scaling reduces the combination of descriptors into 3D space in a nonlinear fashion. Scatter plots in this 3D space show clear, time-dependent segmentation of adipogenic and osteogenic differentiation on non-treated glass (**o**–**r**), which can be observed after 72 h (**q**)^147^. Microtubules mediate nuclear deformations, invagination, volume increase (**s**, **t**) through imposing constraints on the swelling nucleus during hPSC early differentiation into myeloid progenitors. This can be monitored within 24–72 h and quantified via 3D reconstruction of confocal images^120^.

Advances in fluorescence tagging and imaging of molecular markers in live cells have contributed greatly to deciphering multiscale mechanisms underpinning dynamic cell behavior and lineage commitment^6,7,82^. Laser confocal and multiphoton microscopy enable quantification of biological structure–function relationships by quantifying the associated intensity of fluorescence markers or tags of emergent and established changes in cells and their microstructures, e.g., through related gene or protein expression, across space and time scales relevant for tissue development and healing (summarized in Fig. 5). This thereby helps to elucidate cytoskeletal remodeling during stem cell mechanoadaptation and lineage commitment, bridging stem cell structure–function relationships and their clinical translation for regenerative medicine. Versatile protocols for live tracking of cytoskeleton components, using Bacmam® or LifeAct® expression, enable the measurement of actin and tubulin and their co-movements, as well as the measurement of changes in shape and volume of cells and their nuclei, all of which are indicative of structural adaptation^6,143^. Live imaging and quantification of cytoskeleton simultaneous to the introduction of volume and shape-changing stresses (i.e., seeding density and flow) demonstrates the emergence (within the first hour) of anisotropy in the spatiotemporal distribution of actin and tubulin, both with respect to cell polarity (apical to basal) as well as with respect to flow exposure (Fig. 5d–f)^6^. Time-lapse imaging is useful to visualize cytoskeleton dynamics within the first hours of proliferation, indicative of actin and tubulin’s reciprocal influence during neurogenesis (Fig. 5a–c)^144^. In addition, live imaging enables quantification of cytoskeletal crosstalk that occurs within minutes during wound healing^111^ as well as visualization of stem cell recruitment and homing during tissue healing (e.g. using non-invasive in vivo multimodal imaging)^145^. Live imaging thus presents a feasible approach for monitoring cellular and cytoskeletal remodeling over time and in near real time, although gene-based expression for cytoskeleton labeling requires initial optimization to address various transduction efficiency and permeability issues^143^.

Fixed cell imaging shows similar utility for reporting cytoskeletal changes associated with early timepoints in the differentiation process, before changes in baseline gene expression are observable. By pairing high data acquisition confocal imaging with computational analysis, the de-/polymerization of the cytoskeleton can be assessed at pre-determined timepoints. Through work flows, including serial processing and demarcation of images (i.e. filtering, enhancement, thresholding, and segmentation) and structural features (i.e. shape, intensity - indicating degree of formation, location and distribution), reveal changes in actin conformation as early as 24 h on fibronectin-coated glass and 72 h on untreated glass^146^. Such clear segmentation of cytoskeleton descriptors help discern substrate-governed and time-dependent early lineage divergence of hMSCs^146^ (Fig. 5k–r).

Coupling flow cytometry and fluorescence imaging of phalloidin labeled actin highlights distinct filamentous actin patterns in osteogenic and adipogenic differentiating cells within 24 h^130^. This allows the measurement of actin polymerization as a function of early lineage specification ahead of changes in gene expression^130^. In addition to adherent cells, flow cytometry of nonadherent hematopoietic progenitor and stem cells (HPSCs) enables the sorting of populations of CD33 + or CD19 + expressing cells, indicative of respective myeloid or lymphoid progenitor lineages^120^. Coupling this data with confocal imaging and morphometric analysis reveals that nucleus volume and curvature serve as powerful descriptors to discern HPSCs’ early differentiation towards myeloid or lymphoid pathways^120^. Myeloid progenitor cells exhibit significantly larger nucleus volume and invaginations/deformed areas compared to their stem cell and lymphoid counterparts, whereby microtubules bundle together with their centrosome around the nuclear membrane and localized within invaginations. Within 24–72 h of myeloid induction, microtubule constraints generated from dynein motor-associated pulling forces shape nuclear deformation, accompanied by loss of Lamin B IF of the nuclear envelope^120^. 3D reconstruction of DAPI stained nuclei images (Fig. 5s, t) enables quantification of volume expansion and deformed nucleus curvature, link microtubule forces and protective Lamin B filaments in the governance of nucleus shape and heterochromatin distribution during early HPSC differentiation.

Fixed cell imaging is the current standard for the measurement of cell structure using immunofluorescence-based intensity measurements, but fixed cell imaging is not free of limitations. Cell fixation imparts changes in nucleus shape and volume^7^, which can also exert anisotropic shrinkage of cytoskeletal components^7^; hence chemical fixation may introduce artifacts to shape and volume data which underscores the need for new and better methods to observe and measure changes in live cells, and in near real-time. A combination of live and fixed cell imaging can predict early MSC differentiation tendencies by enabling the monitoring of actin turnover^83^ (Fig. 5g–j). Fluorogenic SiR-Actin (a probe based on product of jasplakinolide natural binding to F-actin) binds endogenous actin at every consecutive monomer and decays as soon as a new filament is synthesized. Live imaging that captures the rate of SiR-Actin decay carries information on how rapidly actin polymerization takes place, and the subsequent imaging of phalloidin labeled actin represents the amount of newly synthesized actin^115^. At timepoints as early as 1 h after chemically induced differentiation, this combined imaging method enables the discernment of MSC lineage divergence into osteogenic, adipogenic or chondrogenic populations. New imaging methods offer increased temporal fidelity in the detection and monitoring of stem cells during differentiation and will provide invaluable tools for both fundamental studies of stem cell mechanoadaptation and its clinical translation. Future imaging standards will enable near real-time assessment of structure and function in live cells.

## Materials control of cytoskeletal remodeling

The engineering of synthetic or natural materials to provide biomimetic environments for cells or tissues creates new avenues to study cell behavior and to control complex biological parameters across length- and time scales. Using materials engineering at nano-/micro-scales to modulate cell–matrix interactions, one can probe for cytoskeletal tension to determine structure–function relationships and a wider range of responses, from the cell to the tissue length scales. Patterning of adhesion complexes enables delivery of precise positional cues that can dictate cell shape and geometry by virtue of defining cell adhesion points on substrates^127^. Nanostructures (e.g., pillars, pits, or grooves) provide similar adhesion points and can also be tuned to provide specific local mechanical stiffness mismatch at the interface with the cell. With limitless possibilities and dimension in materials engineering to provide geometric and adhesion cues, along with mechanical parameters like stiffness, could induce an array of cytoskeleton dynamic responses. Therefore, mapping the cytoskeleton adaptive behavior and how it drives lineage commitment may inspire the development and design of next-generation materials and/or devices for tissue engineering and regenerative medicine.

### Materials engineering to introduce multiscale rigidity and geometrical cues

Cells interact with extracellular matrix (ECM) through focal adhesions (FAs), and their binding via integrins to short peptide motifs (e.g. the RGD (arginine–glycine–aspartic acid) peptide derived from adhesion proteins) that controls molecular interactions, tension, and thus cell behaviors^147,148^. It has been shown that altering ligand density (e.g., tuning the number of peptides per cluster) influences the cell attachment strength, and with increasing distraction force and certain ligand cluster conditions, it was observed a peak adhesion strength–adhesion reinforcement behavior that highlights the governing interactions between ligand density, ECM rigidity, and the cell adaptive capabilities^148^. These interactions initiate force stabilization, with respect to ECM mechanical properties, that is achieved through regulation of integrins, ion channels and the cytoskeleton^4^.

With stem cells exhibiting 1000-fold greater sensitivity to adapt to local mechanical environment than terminally differentiated cells^5,21^, the significant influence of matrix stiffness on lineage commitment^149^, and the regulatory role of cell shape and cytoskeleton tension in lineage commitment^27^, the impetus grows to further link cytoskeleton remodeling underpinning stem cell responses to various microenvironmental cues to multiscale materials engineering techniques. Contemporary fabrication techniques to create micro-/nanoscale patterns such as patterning of cell–cell adhesion proteins on solid-supported lipid bilayers^127^, microcontact printing^121,150,151^, photomasking^152^, and lithography^29,153^ have enabled control of cell–materials interaction parameters and elucidation of key mechanisms governing cytoskeleton remodeling, cellular adhesion, migration and differentiation, as well as emergence of multicellular architectures. Transferring patterns of adhesive proteins (e.g., fibronectin) onto a substrate via microcontact printing, enables the introduction of size, shape and geometrical cues to modulate a range of cellular processes and reveal cytoskeleton mechanism in governing cell shape optimization^27,121,154^. Through micropatterning fibronectin on polydimethylsiloxane (PDMS) substrates to create “protein islands” of controlled size, hMSCs’ show contrasting differentiation behavior towards adipogenic or osteogenic lineages. Adhesion onto the small (1024 μm^2^) or large (10,000 μm^2^) islands mimics rounded or spread morphologies that manifest cellular form and function preceding differentiation^27^.

Furthermore, through the introduction of V, T, or Y shape adhesion patterns, cells are confined to achieve steady-state position and cytoskeletal tension by developing concave, non-adhesive edges that connect adhesive points^155^. The presence of one, two or three concave edges observed on the V, T, or Y shaped cells, respectively, is due to actin reinforcement that enhances actin SF strength and renders them more concave upon inhibition of actin polymerization with Y27632^155^. This study demonstrates that actin SF strength depends on the local distance between adhesion sites and correlates inversely to the number of SFs in a cell, globally affecting cell tension. Introduction of square adhesion pattern of different sizes (5–40 μm) also reveals how cells take up the range of size and shape on which they are cultured. This enables the control of cell spreading, where increasing pattern size positively correlates with vinculin focal adhesion concentration^154^. MSCs cultured on patterned substrates of a rectangular shape exhibit higher potential for osteogenesis with increasing shape aspect ratio^121,154^. More complex patterns such as stars, with concave edges and flowers, with convex edges, guide MSC differentiation towards osteogenic and adipogenic lineage, respectively. This correlates with the reorganization of the cytoskeleton to adapt to those contrasting shapes; star shapes present high contractile edges, thicker SFs and larger focal adhesion localization at sharp vertices, while flower shape contains SFs forming contractile point at corners between petals^121^. Star-shaped MSCs exert higher traction stresses on hydrogel substrate in a stiffness-dependent manner than those of circle-shaped^156^. This stiffness-dependent increase in traction stress is observed on fibronectin-coated substrates which enhances α5β1 and αvβ3 integrin expression, as well as osteogenic and myogenic differentiation^156^. The mechanism(s) by which cells adapt to geometric cues (size and shape) through regulated synthesis of actin or microtubules, as well as their distribution and linkage with integrin focal adhesions, indeed dictates the mechanochemical signals directing cell fate.

Actin’s capacity to sense rigidity and to adapt lies in its capacity to sense mismatches in mechanical properties at interfaces with its environment and its capacity to switch from fluid-like to solid-like material as the cell culture substrate rigidity increases^30^. With increasing substrate stiffness, actin SFs form clusters or microdomains within the cell; these clusters vary in orientation (angle), followed by transition of fiber alignment toward a predominant direction at maximum stiffness^30^. The arrangement of actin orientation may reflect the differentiation state of the cell, which could in the future be targeted by fine-tuning material stiffness or surface topographies, down to modulating subcellular traction forces via micropillar height or diameter^157^. Nanopillars provide both positional and mechanical stiffness cues directly to cells, while patterns of adhesive proteins provide direct positional cues which can result in secondary mechanical effects. With shorter heights of silicon micropillar array substrate, the spring constant (reflecting the stiffness) of each pillar increases and thus increases the spreading of cells, SF organization and focal adhesion^132^. On softer, higher pillars, cell area decreases with disorganized, shorter stress fibers, and smaller focal adhesion complexes which supports adipogenic differentiation in hMSCs^132^. Stiff micropillars promote osteogenic differentiation consistent with increased stress fiber thickness and organization, providing a link between biophysical (material) cues and the resulting structure–function relationship^132,157^.

A similar adaptive switch in cytoskeleton properties is observed using a chemically etched nanopillars platform that modulates cell spreading. Nanopillars with different spacing and diameters reduce cell spreading, where cells thus exhibit a rounded morphology. Transition from rounded to extending morphology on flat surface correlates with the adaptive ordering of cytoskeleton represented as a switch in orientation of the filaments. On flat surfaces, actin bundles show larger diameters and exhibit a predominant orientation, while on nanopillar arrays, cell traction forces balance adhesion with basal and peripheral nanopillars, resulting in more diverse orientations of actin^158^. In contrast, microtubule orientation exhibits higher diversity in cells on flat surfaces, as extensive radiality/multidirectionality is required to complement high actin contractility^158^. Mapping these directional responses to the magnitude of biophysical cues could help to elucidate the interactions and counterbalance effects of actin and tubulin, with relevance for tissue neogenesis and pathophysiology.

Cellular mechanisms of rigidity sensing couple tightly with cytoskeletal tension, presenting a feedback loop driven by the half-life of integrin receptors. Integrins are found to be short-lived on softer substrates, thus providing a less stable linkage between actin and the substrate, resulting in a more fluid behavior of actin as well as a higher dynamic in cell behavior. On stiffer substrates, integrins cycle longer and contribute to higher cell membrane order, allowing focal adhesion maturation and providing a more stable linkage with actin^45^. Stability at focal adhesion–actin-mediated adhesions on substrates translates to cytoskeletal force balance during cell–materials interaction that is achieved when adhesion force equals contractility. Intriguingly, this cytoskeletal force balance may also underpin the motility state of the cell (adherent versus motile)^4^.

On simple 3D collagen gels, the early adaptation of fibroblasts involves rapid formation of protrusions with high actomyosin contractility that is counterbalanced with restricting microtubule growth at the tip of protrusions^159^. On nanoscale porous substrates, increasing pore size reduces actin intensity which otherwise spans the entire length of cells grown on nonporous substrates^31^. Actin accumulates in the larger pores and thick stress fibers connect between these pores, covered by a cell. These effects are due to actomyosin tension required to span across the pores and to sustain the increasing stress present in the proximity of the pores and to maintain adhesion^31^. Electron beam lithography fabricated nanotopographies with various patterns of symmetry and disorderliness provide controlled nanoscale roughness that modulates osteogenic differentiation in MSCs^160^. Well-spaced, highly ordered nanopatterns create available surface area with consistent pillar to pit ratios and thus allowing actin SFs to initiate less adhesion points. In contrast, highly disordered, randomly spaced nanotopographies show an improved cell adhesion and defined SFs, leading to significantly increased expression of the osteogenic specific markers, osteopontin and osteocalcin^153,160,161^. Disordered nanotopographies may promote stress fiber polymerization to create adhesion bridging^161^ between spatially random nanopillar adhesion sites and to counterbalance the additional cellular tensile stress to bridge the irregular surface gap size^161^.

Other physical means of guiding cytoskeletal spatial organization such as roughness, grooves/ridges, or curvatures at micro or nanoscale provide links between surface topography and topography-induced cell processes and tissue formation^158,160,162,163^. From a study using native rat dental tissue slabs with rectangular grids of *circa* 350-μm wide grooves as a template for osteoblast culture, it was first reported that macroscale bone formation occurs within 2 weeks and that local topography plays an important factor in determining spatiotemporal tissue characteristics^163^. Specifically, newly formed bone initiates focally and aligns linearly along the grooves, with more pronounced and total mineralization in deeper trenches by day 18. A high proportion of osteocytes also align along the groove axis and form rings along the rim of the tissue slab, where growth outside the grooves occurs after day 19^163^. Nanotopographies introduced as nanopits and grooves with varying width and depth explain cytoskeleton contact guidance behavior to groove and pit edges initiated by filopodia. Increase cell spreading is observed in all pit sizes with defined stress fibers and increased microtubule network density. On smaller width grooves, both actin and microtubules align and condense along the narrow ridges showing higher filament density that correlates with higher osteocalcin and osteopenia expression than those on nanopits^160^. The constraints to marginal expansion, provided by the narrow grooves promote defined cytoskeleton alignment and larger vinculin focal adhesion in the groove direction, all contribute to osteogenic functionality. Use of SiO_2_ nanostructure with controlled membrane curvature results in the accumulation of F-actin and actin polymerization initiator, ARP2/3 and Formin, around the nanopillar structure^29^. Similarly, cells cultured on microcontact printed circle patterns of 3–5 μm diameters show accumulation of focal adhesion complexes (vinculin, talin, and integrins) around the circle curvatures^154^. Focal adhesion concentration increases with decreasing circle diameter as it promotes maximum cell spreading whereby SFs are required. In line with the requirement of an actin template for focal adhesion maturation^63^, this curvature dependency of actin localization indicates a potentially broader reorganization mechanism of actin, that is to ensure high actin polymerization as focal adhesions localize and mature along the curvature.

### Materials engineering at peptide scale to tune ligand presentation

Finally, engineering ECM-like substrates via immobilization of RGD peptides with tailored ratios and spacing enables one to peer through the molecular machinery of cell adhesions and their effect on cytoskeleton organization and behavior. The use of various aspect ratios of immobilized RGD peptides on gold nanorods supports anisotropic presentation of ligand binding to the cells, in which large aspect ratio increases cell spreading, focal adhesion size, and vinculin intensity^164^. This aspect ratio also modulates cell spreading and adhesion via recruitment of integrin clusters, measured by mechanotransduction protein, YAP, nuclear localization and size of focal adhesion kinase (FAK)^164^. Engineering nanoscale interfacial roughness gradient by tilt dipping substrate in self-polymerized, fast-deposited cathecholic polyglycerol solution results in increase in roughness (95% polymer composition) that reduces cell spreading. High roughness regions also reduce the order and bundle size of actin stress fibers; as well as area and length of FA protein, Paxillin, which otherwise peaks at 50% roughness^165^. Gradual increases in osteogenic differentiation occur across a roughness gradient, reaching a maximum at 50% roughness and dropping at a higher roughness area (>75%) where adipogenesis is promoted^165^. How interfacial roughness is sensed through actin ordering, focal adhesion maturation and Paxilin recruitment reveals the optimum roughness at which cells show the maximum capacity to differentiate and to adapt to increasing cellular and nuclear tension^165^. Presentation of spatially ordered and disordered RGD nanopatterns provides a possibility for decoupling global RGD density with local integrin clustering and elucidates the ligand spacing threshold within which stable cell adhesion can be “turned on or off”^166^. Cells cultured on disordered nanopatterns present higher spreading and thicker actin bundles than those on ordered patterns, due to the polydispersity and wide variation in ligand spacing^166^. The stable attachment and spreading indeed are more likely regulated through local ligand density including clustering of ligands and their spacing, than the global available functionalized ligands^166,167^. Modifying the ratio of RGD peptide and ethylene oxide elements (EO_6_, to prevent non-specific adhesion) on silicon surfaces could achieve various spacing of RGD peptides that serve as the only factor controlling adhesion^168^. Across the range of RGD:EO_6_ ratio on surfaces, optimum cell number and spreading occur with 44 nm RGD spacing. Endothelial cells cultured on this spacing show an enhanced FAK phosphorylation and ERK1/2, AKT signaling which centrally regulates angiogenesis^168^. Tailoring ligand arrangement and presentation to cells provide a means to isolate the complex and coupled physiologically relevant ECM cues such as stiffness, composition and ligand concentration; and to understand their implication in many biological functions such as differentiation, proliferation and vascularization, through focal adhesions and cytoskeleton behavior.

Taken together, the motivation to understanding cell or tissue behavior necessitates the integration of interdisciplinary approaches to engineering micro- or nanoscaled materials that provide signaling cues mimicking natural matrices. Exposing cells to controlled geometric, topographic and molecular cues has progressively revealed fundamental insights into underlying mechanisms, from the smallest scale of adhesive protein interaction to the genesis of larger, multicellular tissue templates. The function of actin and microtubule cytoskeleton function in regulating traction forces, contractility, and compressibility of cells translates to their rate of de-/polymerization and spatial distribution conducive to guiding lineage commitment. The unique cytoskeleton responses, brought about by micropatterns to nanoarrays of various shape, size, and spacing, reflect structural adaptation that bridges the interplay of those complex, multidimensional biophysical cues. Linking and mapping these findings across time- and length scales could help address the remaining challenge to recapitulating tissue niches and nature’s own design templates that mimic the biointerface and intrinsic functionality of ECM.

## Outlook

The cytoskeleton represents a living structure that couples the interior and exterior of the cell, conferring to cells the capacity to respond to biomechanical, -chemical, and -physical stimuli in their environment as well as to move, probe and grow into surrounding tissues. By virtue of providing cells with physical scaffolding that responds to dynamic needs of the cell, like a “living bridge”, the cytoskeleton constantly adapts and reconfigures and balances forces at the intersection between the cell and its environment; in the process, the cytoskeleton literally shapes the cell and influences its own environment via up- and down expression of structural proteins and other constituents of the extracellular matrix. As a whole, the ubiquitous effects of cytoskeletal remodeling in response to biomechanical, -chemical, and -physical cues, confers robustness and resilience to cells, enabling adaptation and increasing survival under dynamic enviromental conditions.

The complex regulatory processes and effects of stem cell mechanoadaptation comprise a balance of endogenous and exogenous biophysical and chemical forces whereby the mechanical and chemical milieu of the cell literally shapes the cell and drives its differentiation and the emergent development of tissues with specific structure and function. The cytoskeleton is the major shape regulatory and mechanoadaptive element of the cell and as such is not only a valuable indicator but also a target for a range of cellular and tissue functions in health and disease. Targeting cytoskeleton dynamics via the introduction of controlled chemical, mechanical or biophysical cues will provide valuable insights to tissue development across time and length scales; this may in turn enable one to track or predict specific developmental stages that can be harnessed to improve targeted tissue neogenesis or build relevant disease models. Mechanomics describes the interrelationship between the mechanical environment and the adaptation of biological entities in response to changes in that environment; at the length scale of a stem cell mechanomics describes the role of mechanics on the emergent expression of structural proteins. From a stem cell to an organismal perspective, mechanomics is key to understanding the complex cytoskeletal responses to controlled mechanical cues across scales whereby factors affecting single cells (individually) could be compensated for or canceled out in the context of larger scale multicellular constructs (globally). Through bridging of disciplines in chemistry, physics, biology, and mechanics of the cytoskeleton, and through the use of experimental and computational tools, it will be possible to decipher cytoskeletal control of shape and fate. This knowledge can be harnessed to scale up engineered tissue templates and to improve design of materials or next-generation devices (e.g. surgical implants, mechanoactive tissue engineering scaffolds) for drug delivery or regenerative medicine.
